# Supplementary material for: Human pannexin 1 channel is not phosphorylated by Src tyrosine kinase at Tyr199 and Tyr309
Source: eLife. 2024 May 23;13:RP95118. doi: 10.7554/eLife.95118 (PMC11115448; doi:10.7554/eLife.95118)
Supplement: Figure 2—source data 1. [file elife-95118-fig2-data1.zip › Figure 2-source data 1/figure2_source_data_1.pdf]

# Figure 2-source data 1

Upper panel of Figure 2B

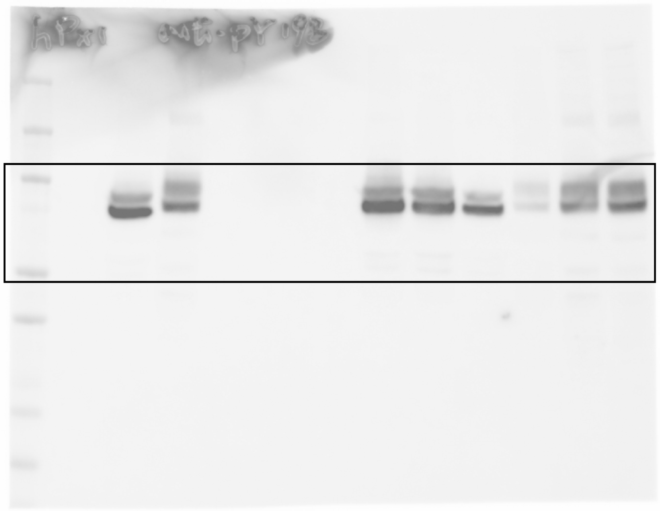

anti-PANX1-pY198

Middle panel of Figure 2B

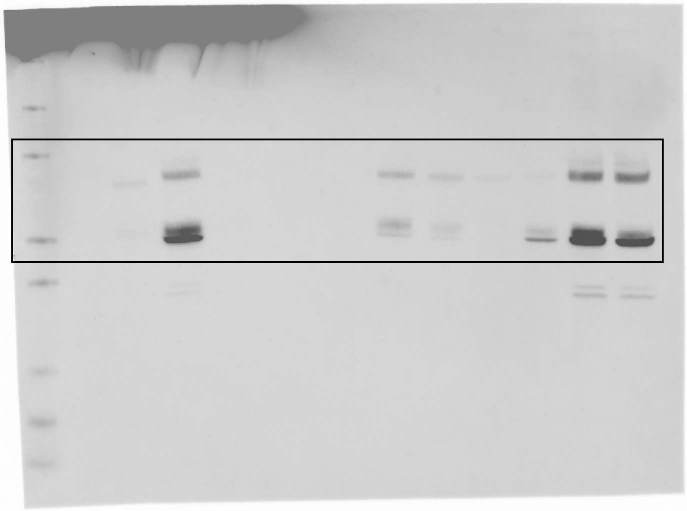

anti-PANX1-pY308

Lower panel of Figure 2B

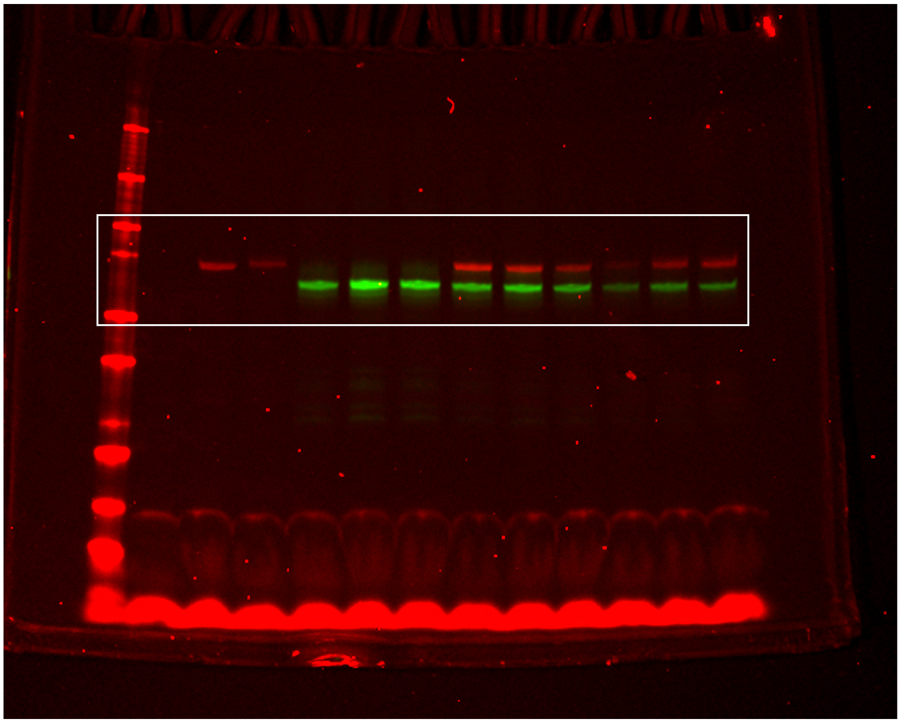

GFP & mCherry fluorescence
