# Supplementary figures and images for: Human pannexin 1 channel is not phosphorylated by Src tyrosine kinase at Tyr199 and Tyr309
Source: eLife. 2024 May 23;13:RP95118. doi: 10.7554/eLife.95118 (PMC11115448; doi:10.7554/eLife.95118)

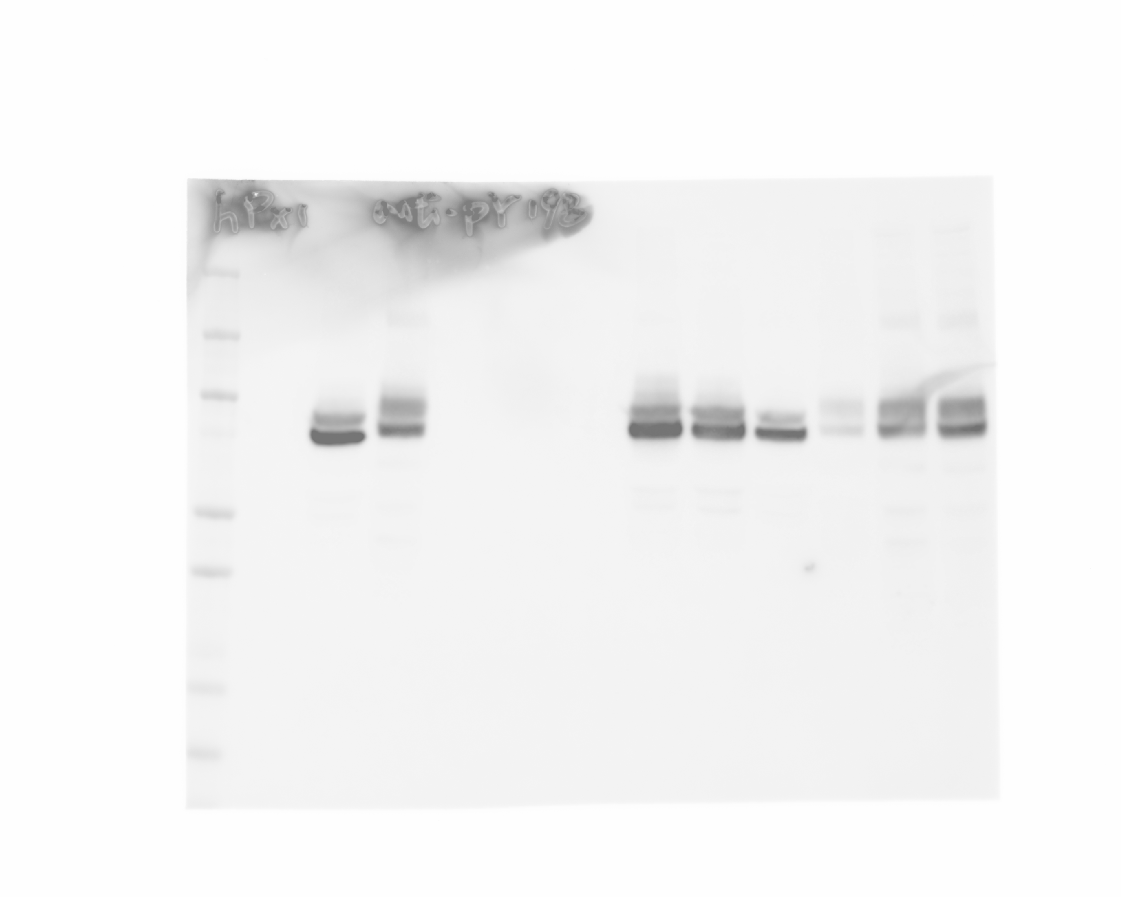

Supplement: Figure 2—source data 2. [file elife-95118-fig2-data2.zip › Figure 2-source data 2/Figure2B_anti-PANX1-pY198 copy.tif]

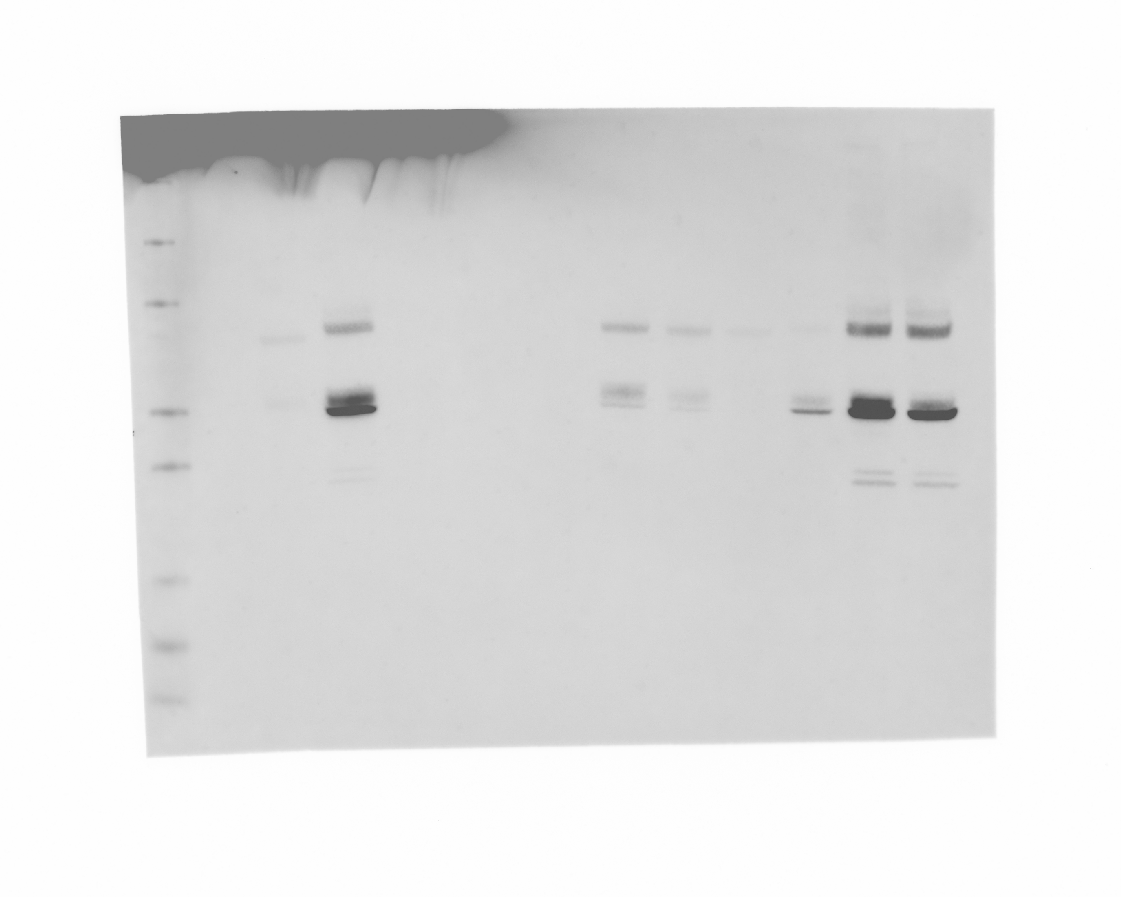

Supplement: Figure 2—source data 3. [file elife-95118-fig2-data3.zip › Figure 2-source data 3/Figure2B_anti-PANX1-pY308 copy.tif]

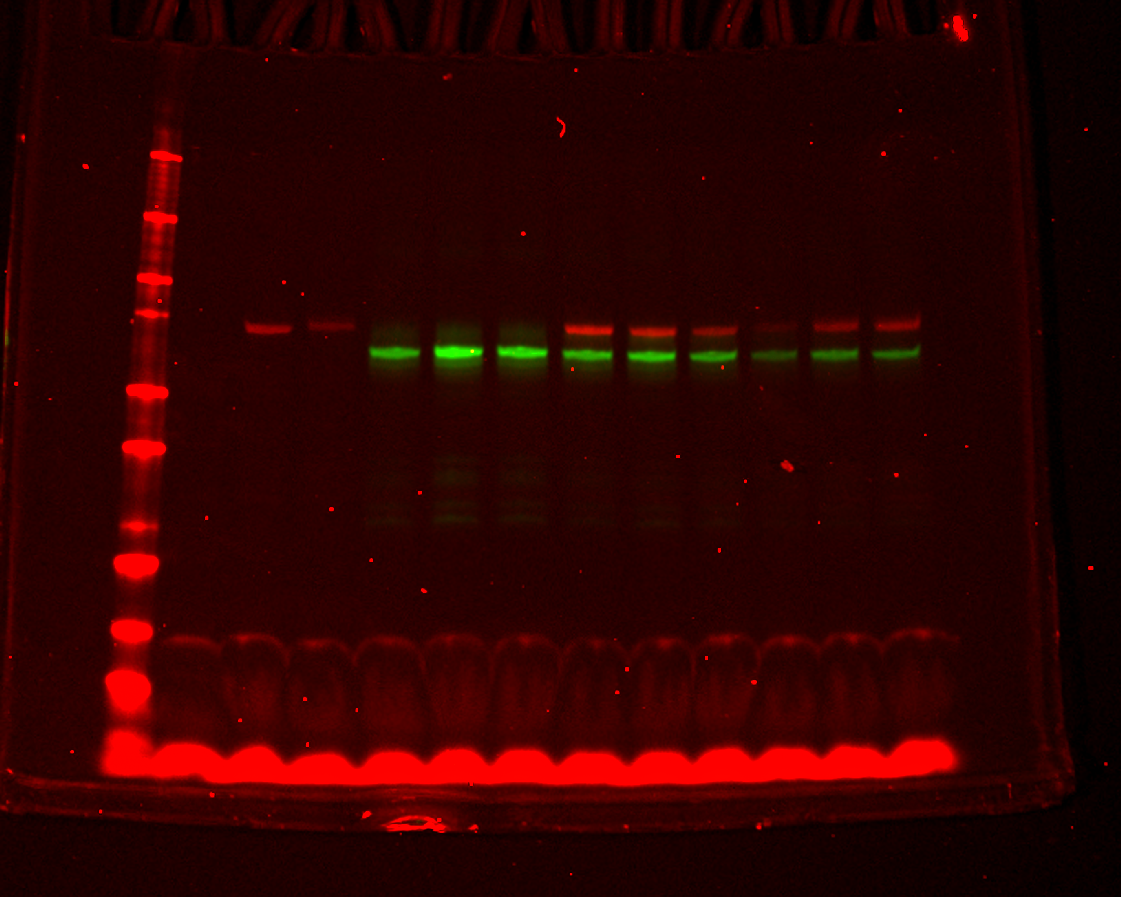

Supplement: Figure 2—source data 4. [file elife-95118-fig2-data4.zip › Figure 2-source data 4/Figure2B_fluorescence copy.tif]

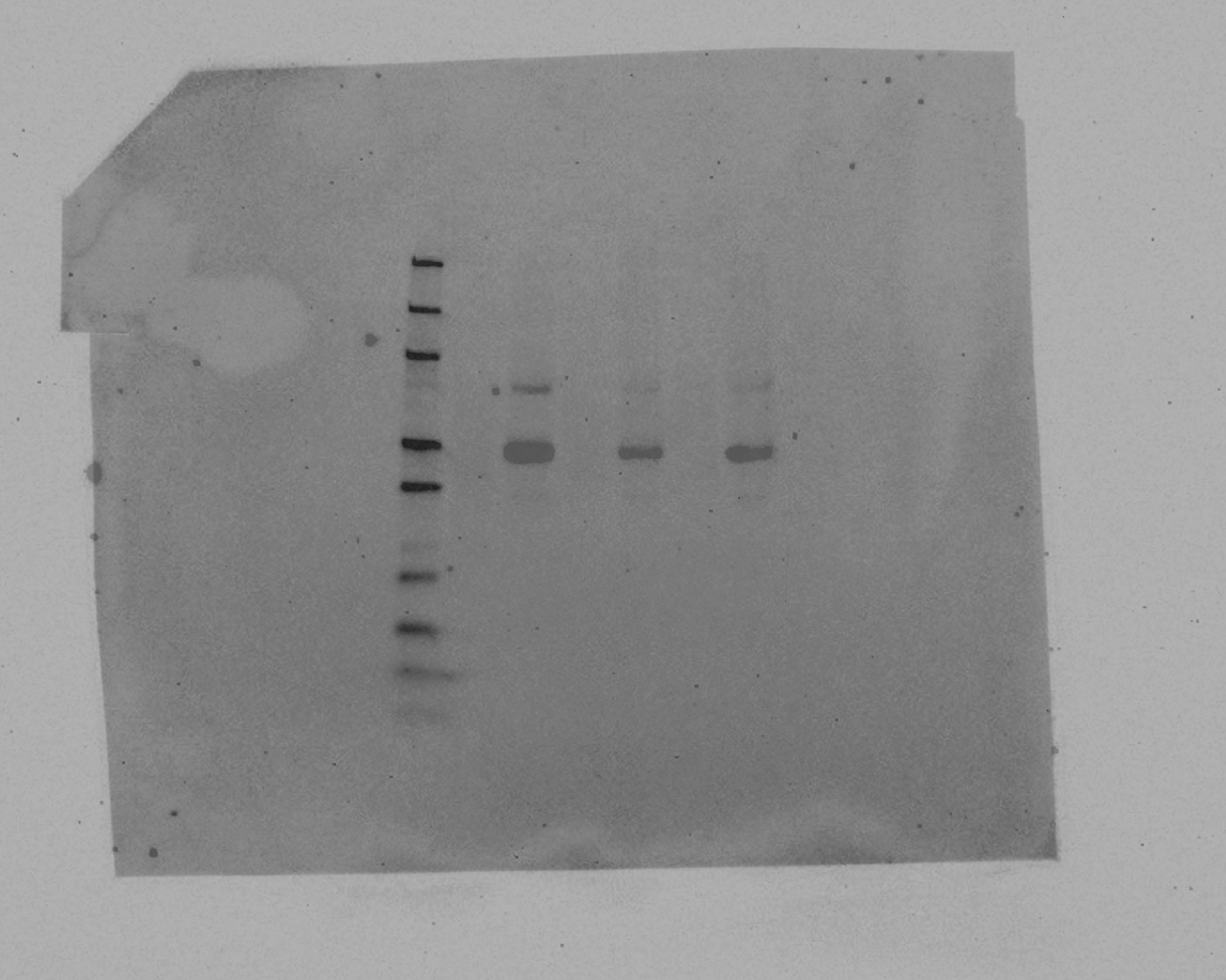

Supplement: Figure 2—figure supplement 1—source data 1. [file elife-95118-fig2-figsupp1-data1.zip › Figure 2-figure supplement 1-source data 1/anti-PANX1-pY308.tif]

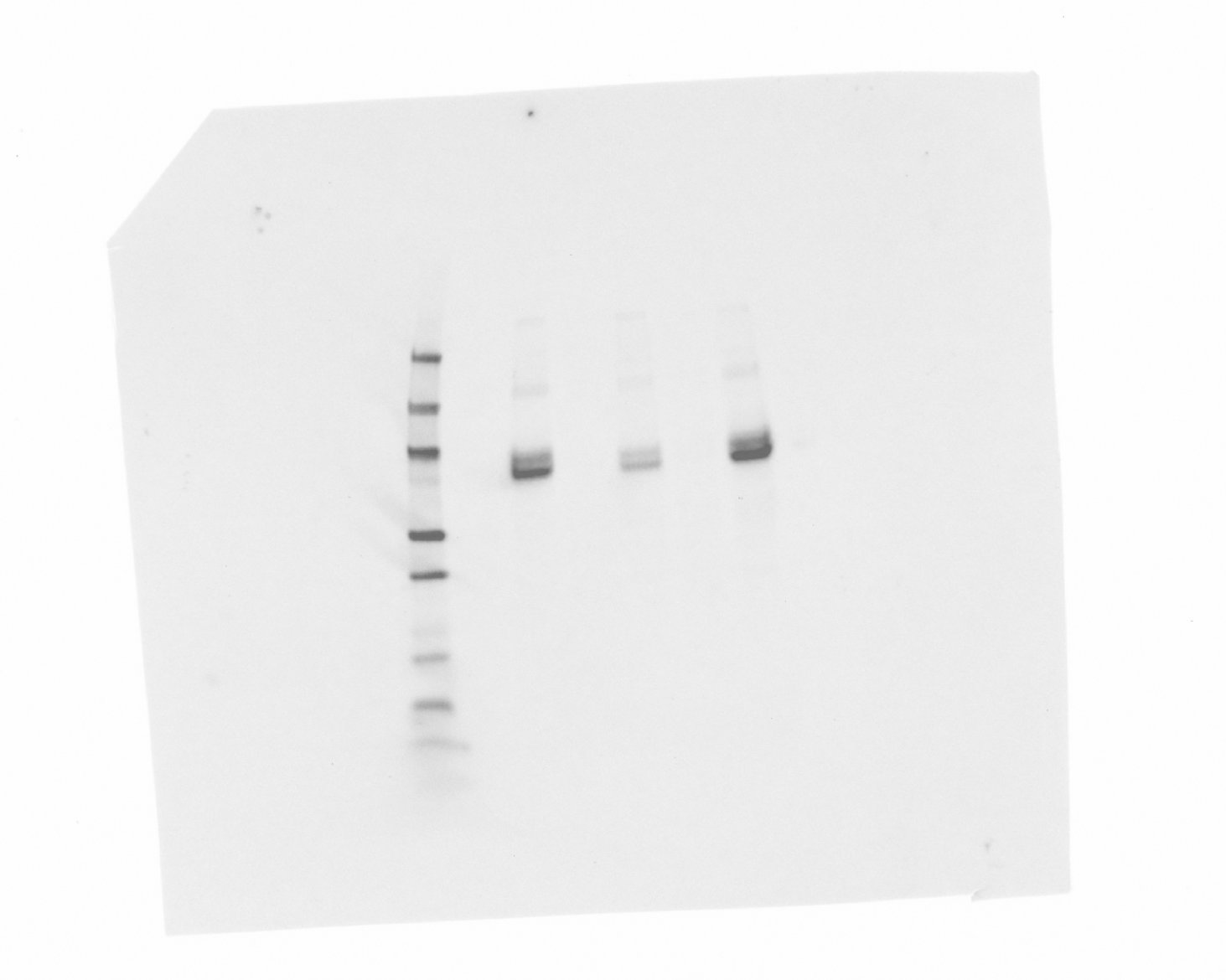

Supplement: Figure 2—figure supplement 1—source data 1. [file elife-95118-fig2-figsupp1-data1.zip › Figure 2-figure supplement 1-source data 1/anti-PANX1-pY198.tif]

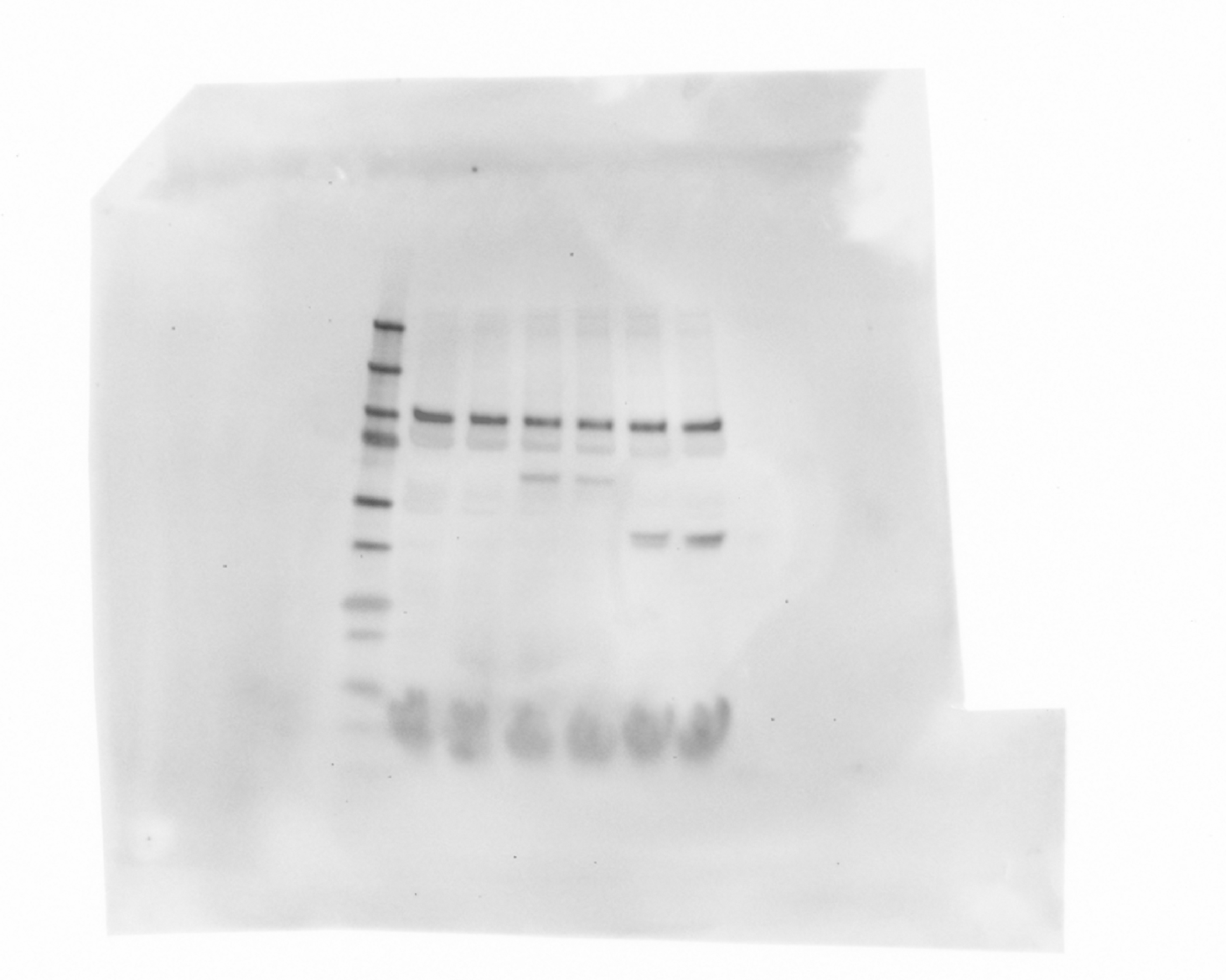

Supplement: Figure 2—figure supplement 1—source data 2. [file elife-95118-fig2-figsupp1-data2.zip › Figure 2-figure supplement 1-source data 2/anti-PANX1.tif]

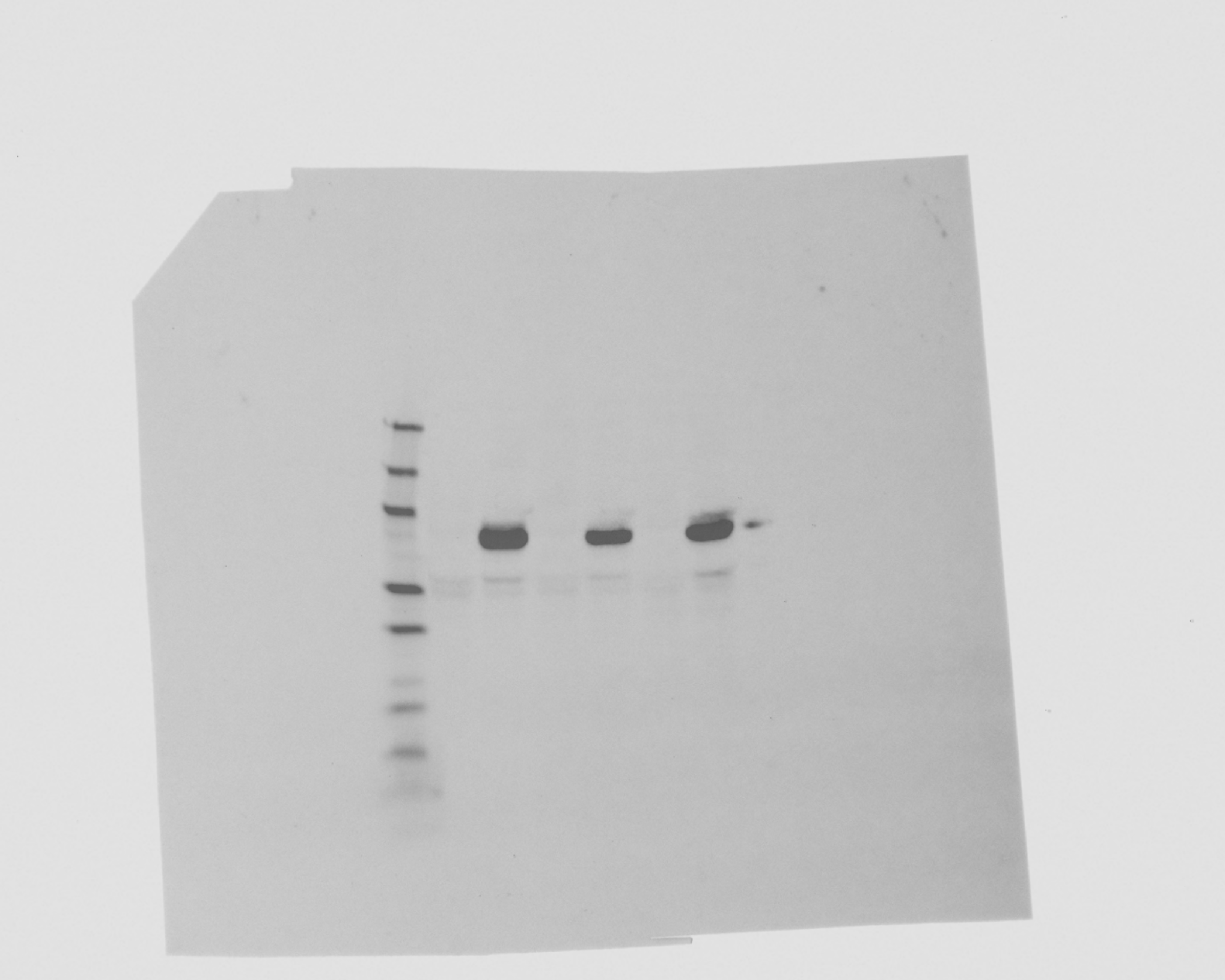

Supplement: Figure 2—figure supplement 1—source data 2. [file elife-95118-fig2-figsupp1-data2.zip › Figure 2-figure supplement 1-source data 2/anti-Src.tif]

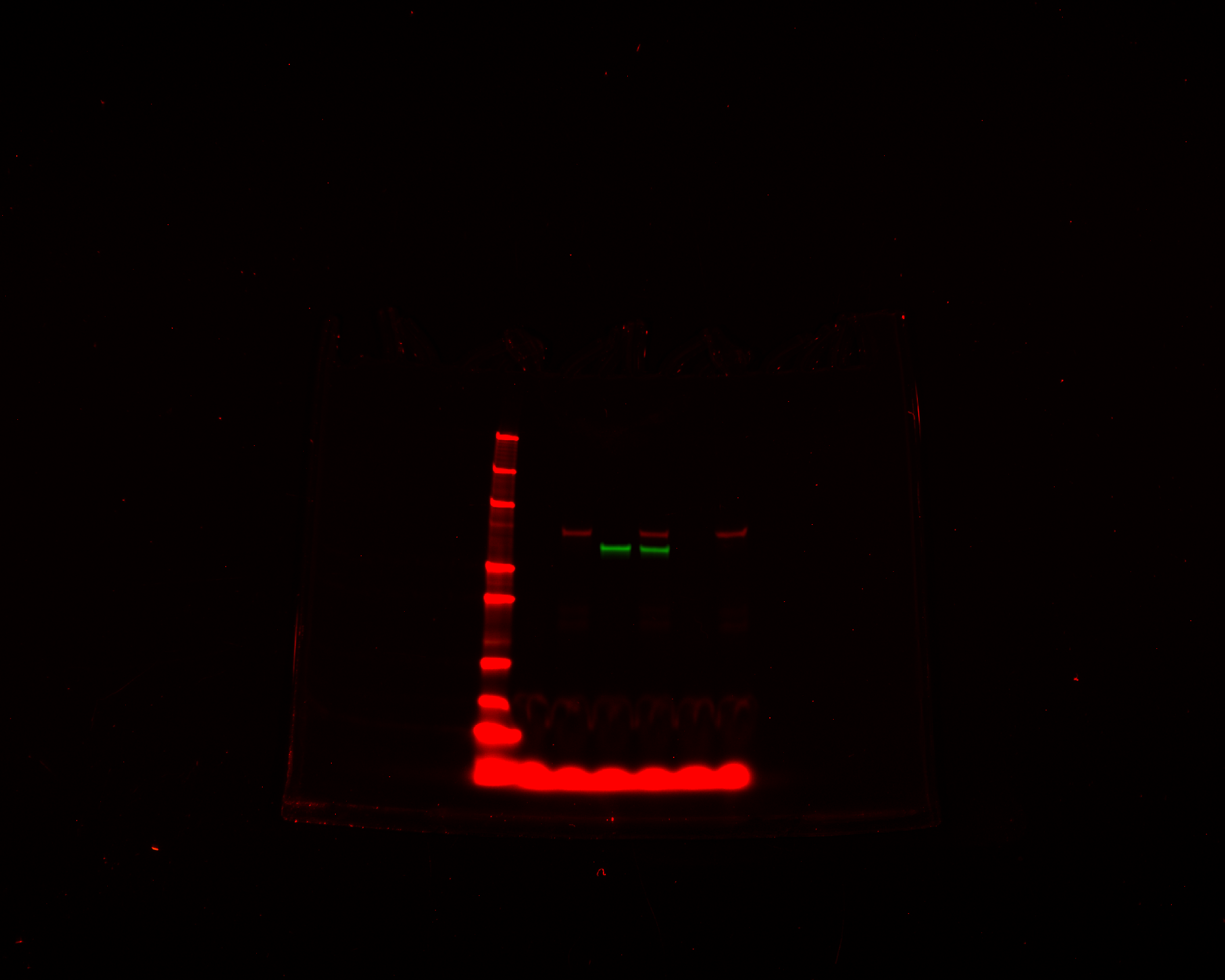

Supplement: Figure 2—figure supplement 1—source data 3. [file elife-95118-fig2-figsupp1-data3.zip › Figure 2-figure supplement 1-source data 3/GFP-mCherry_fluorescence.tif]

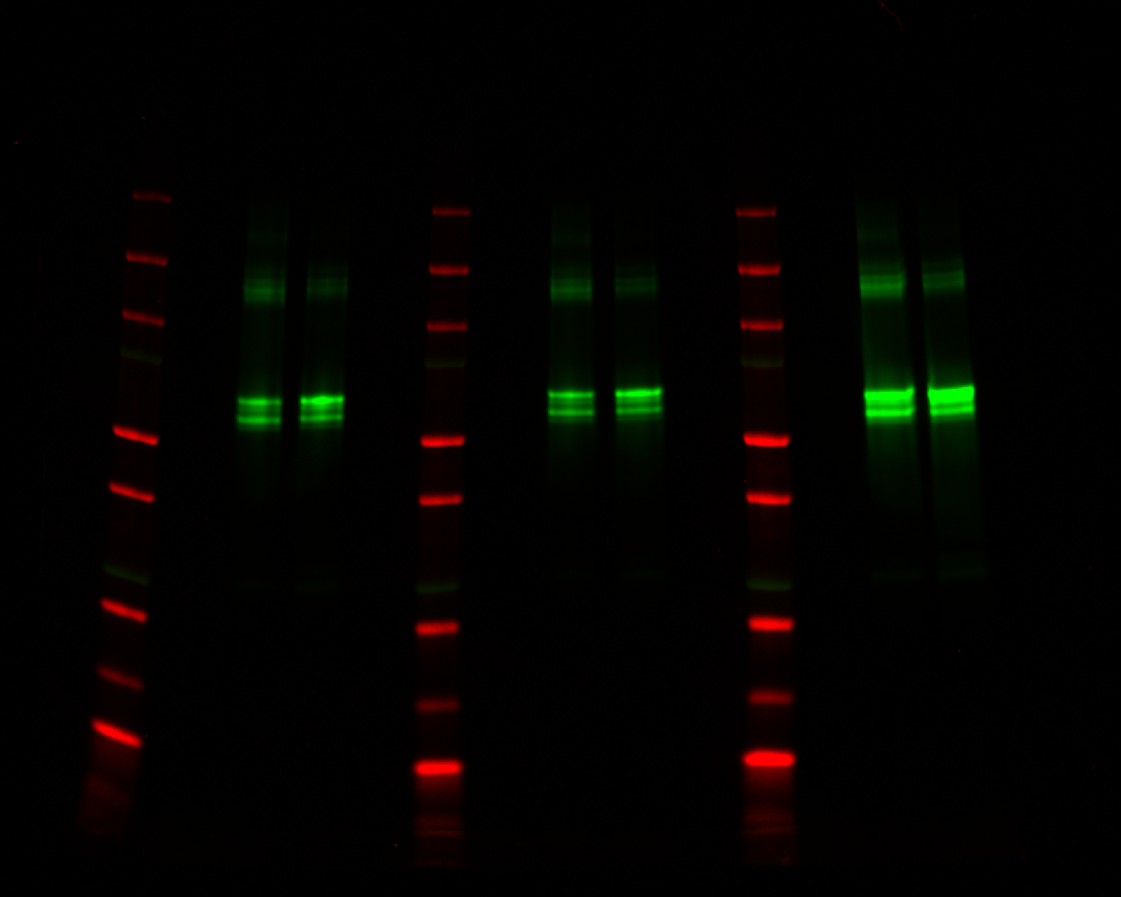

Supplement: Figure 3—source data 2. [file elife-95118-fig3-data2.zip › Figure 3-source data 2/Figure3A_fluorescence.tif]

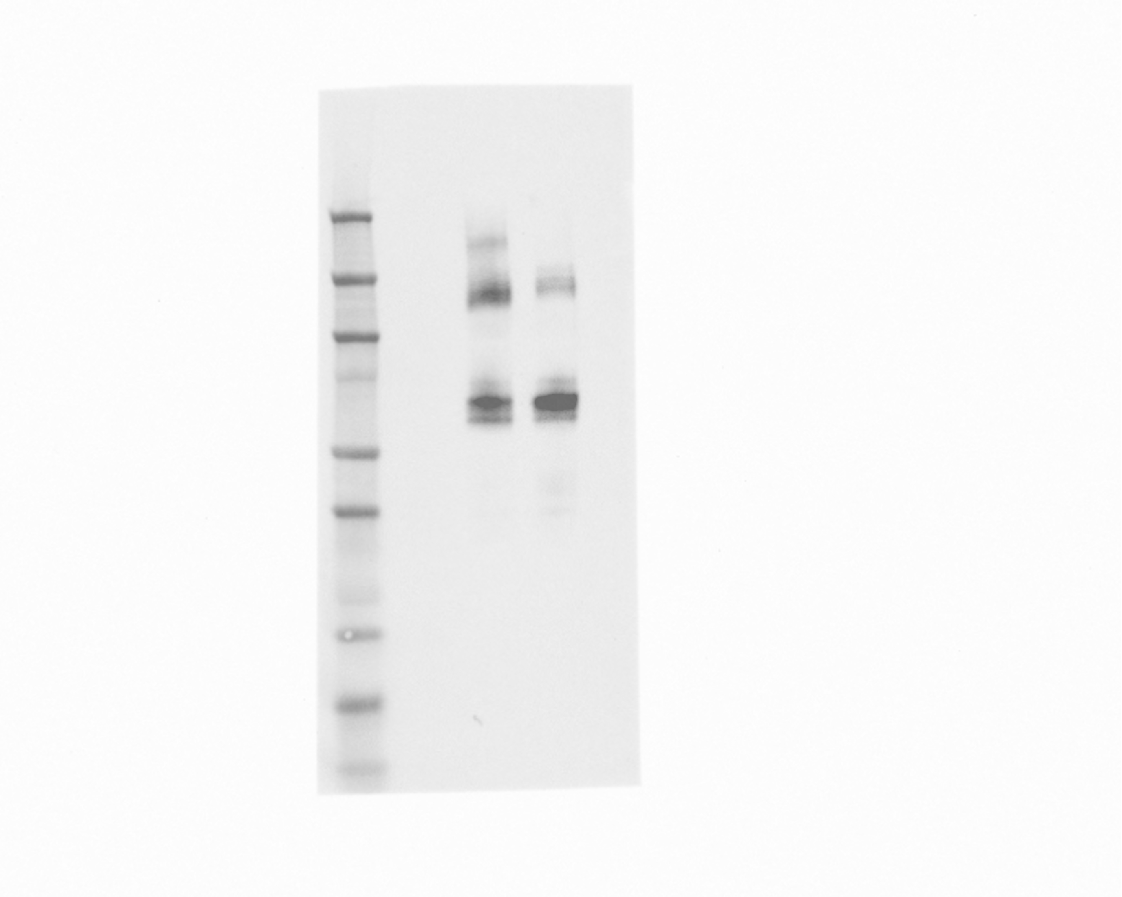

Supplement: Figure 3—source data 3. [file elife-95118-fig3-data3.zip › Figure 3-source data 3/Figure3A_anti-PANX1.tif]

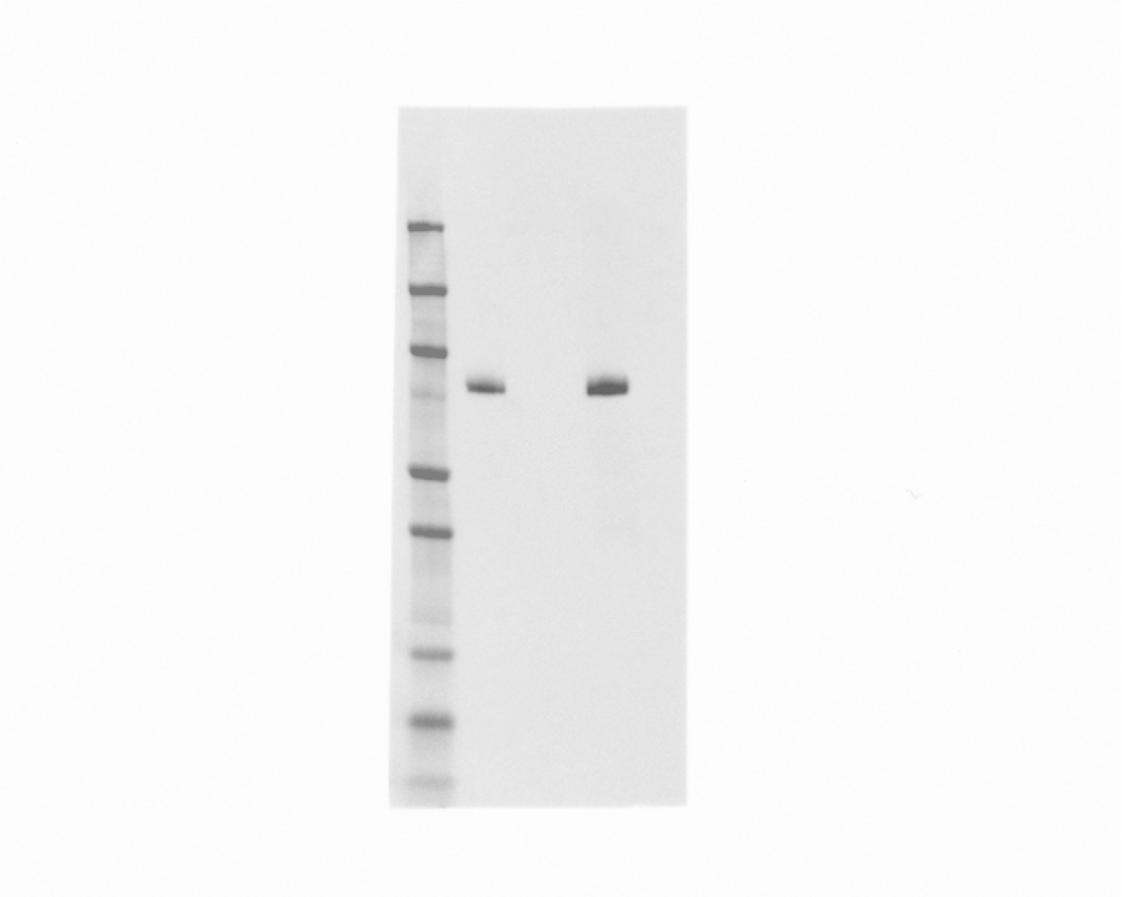

Supplement: Figure 3—source data 4. [file elife-95118-fig3-data4.zip › Figure 3-source data 4/Figure3A_anti-pY100.tif]

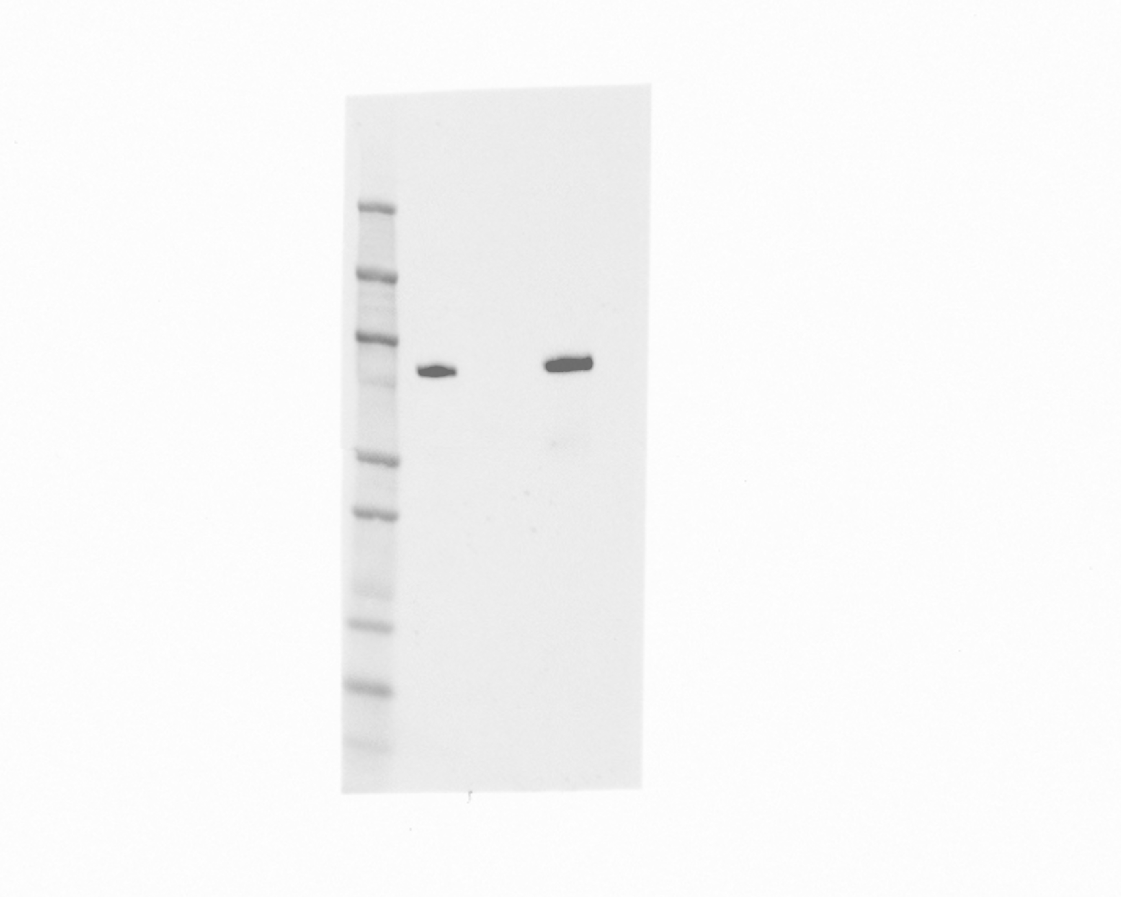

Supplement: Figure 3—source data 5. [file elife-95118-fig3-data5.zip › Figure 3-source data 5/Figure3A_anti-Src.tif]

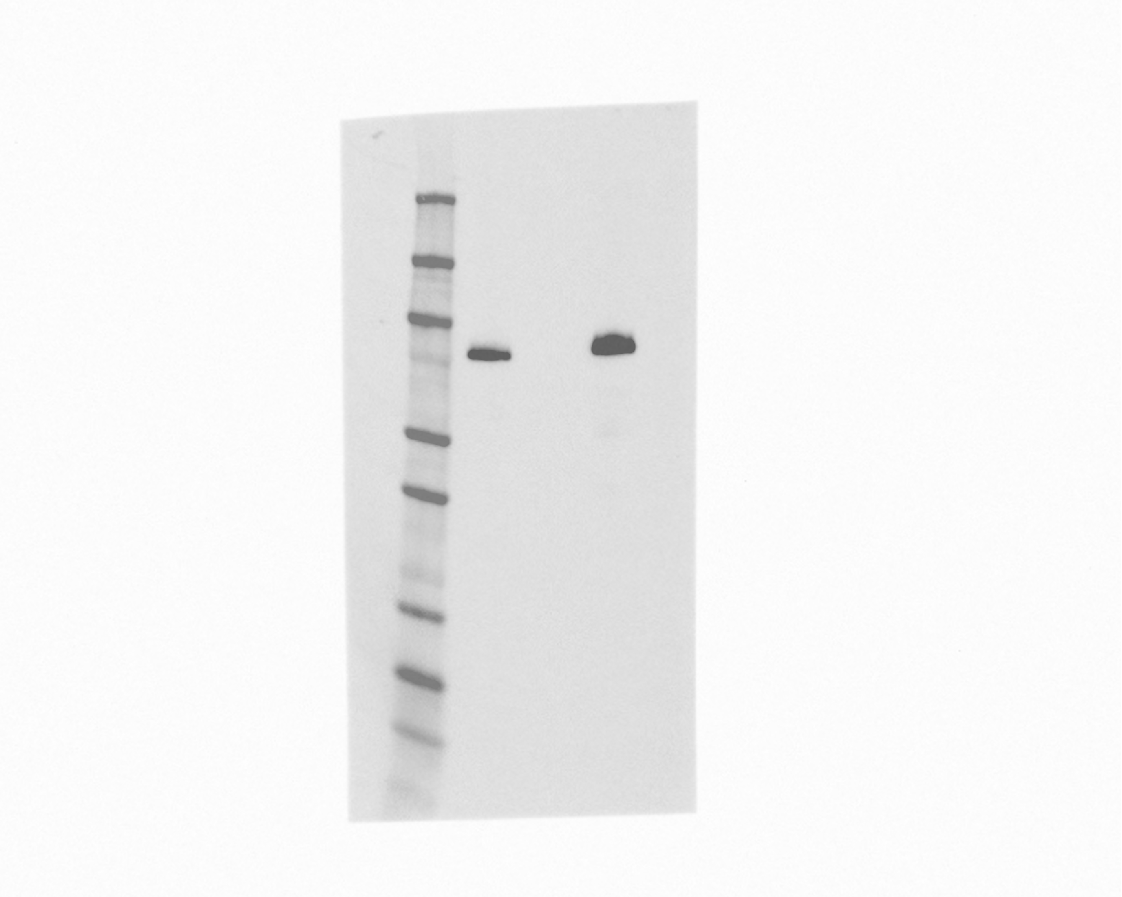

Supplement: Figure 3—source data 6. [file elife-95118-fig3-data6.zip › Figure 3-source data 6/Figure3A_anti-PANX1-pY198.tif]

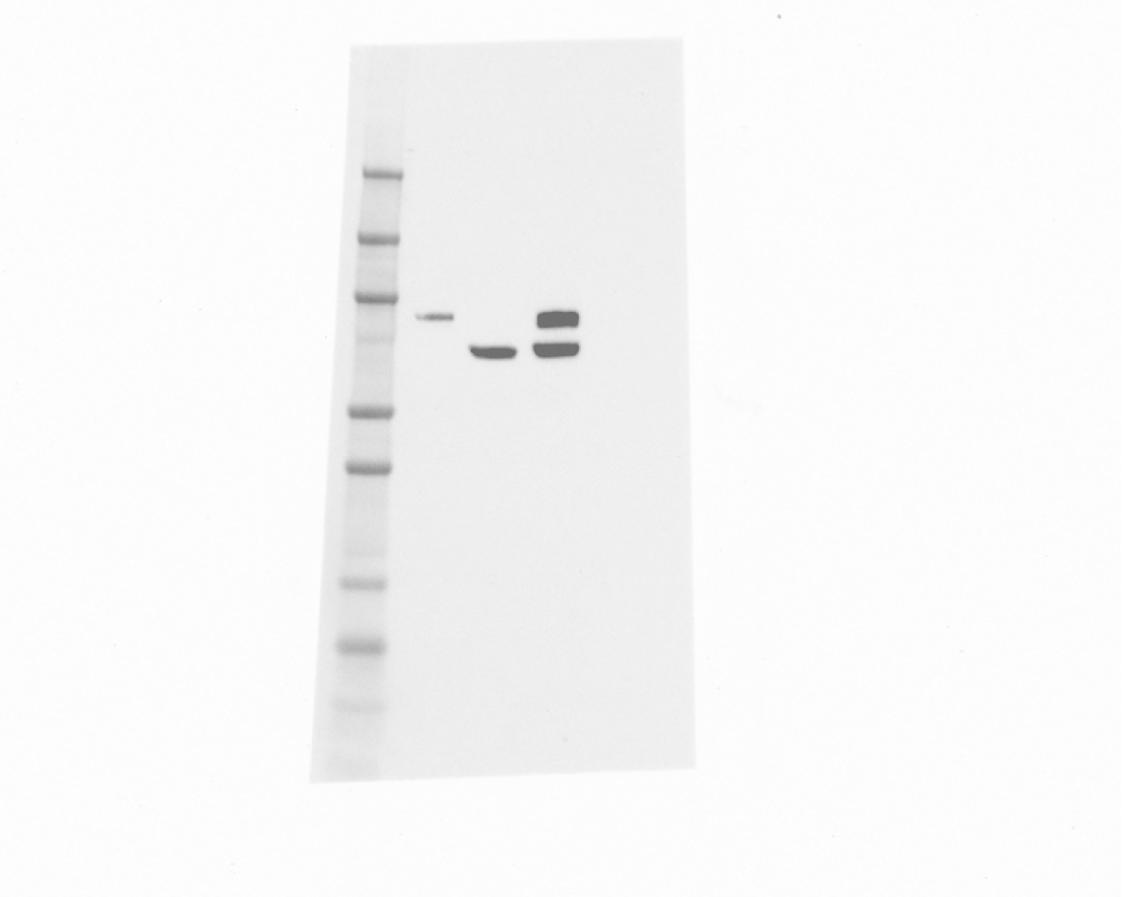

Supplement: Figure 3—source data 7. [file elife-95118-fig3-data7.zip › Figure 3-source data 7/Figure3A_anti-PANX1-pY308.tif]

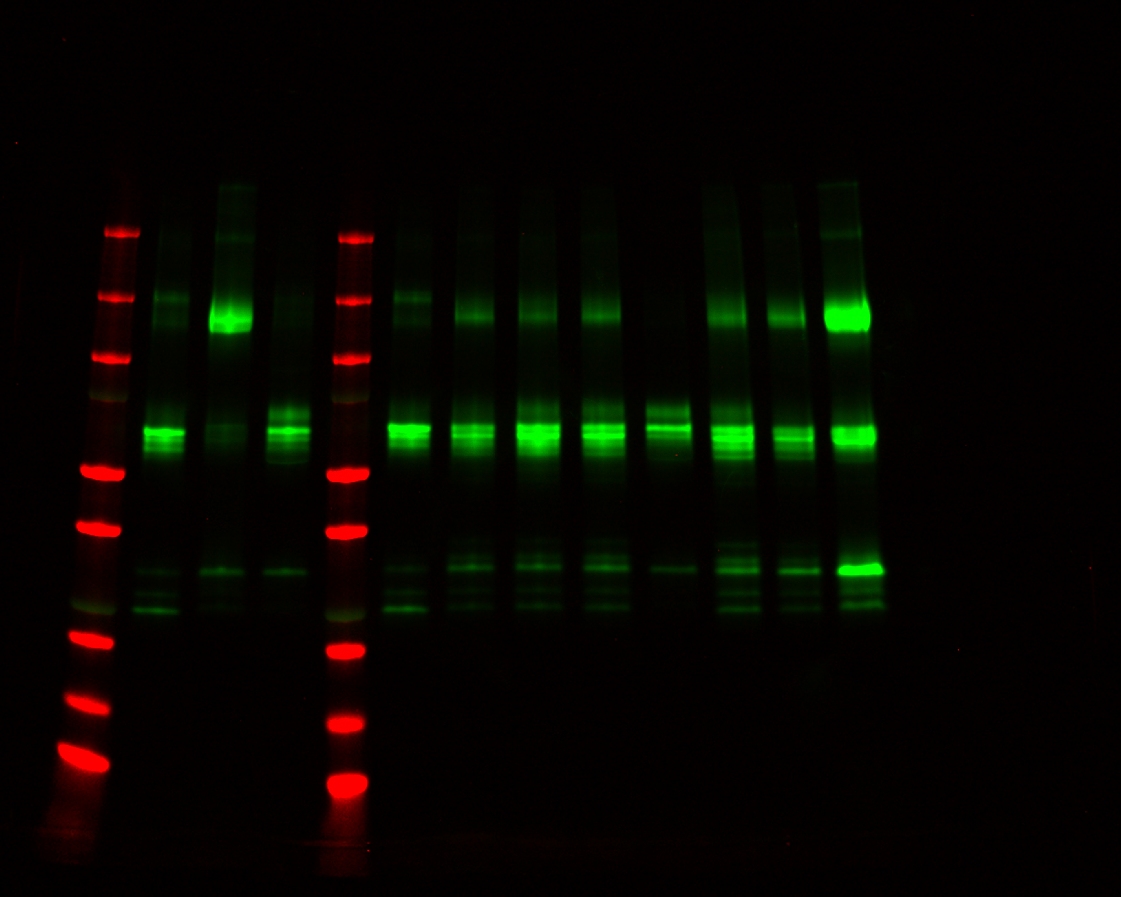

Supplement: Figure 3—source data 8. [file elife-95118-fig3-data8.zip › Figure 3-source data 8/Figure3BC_fluorescence.tif]

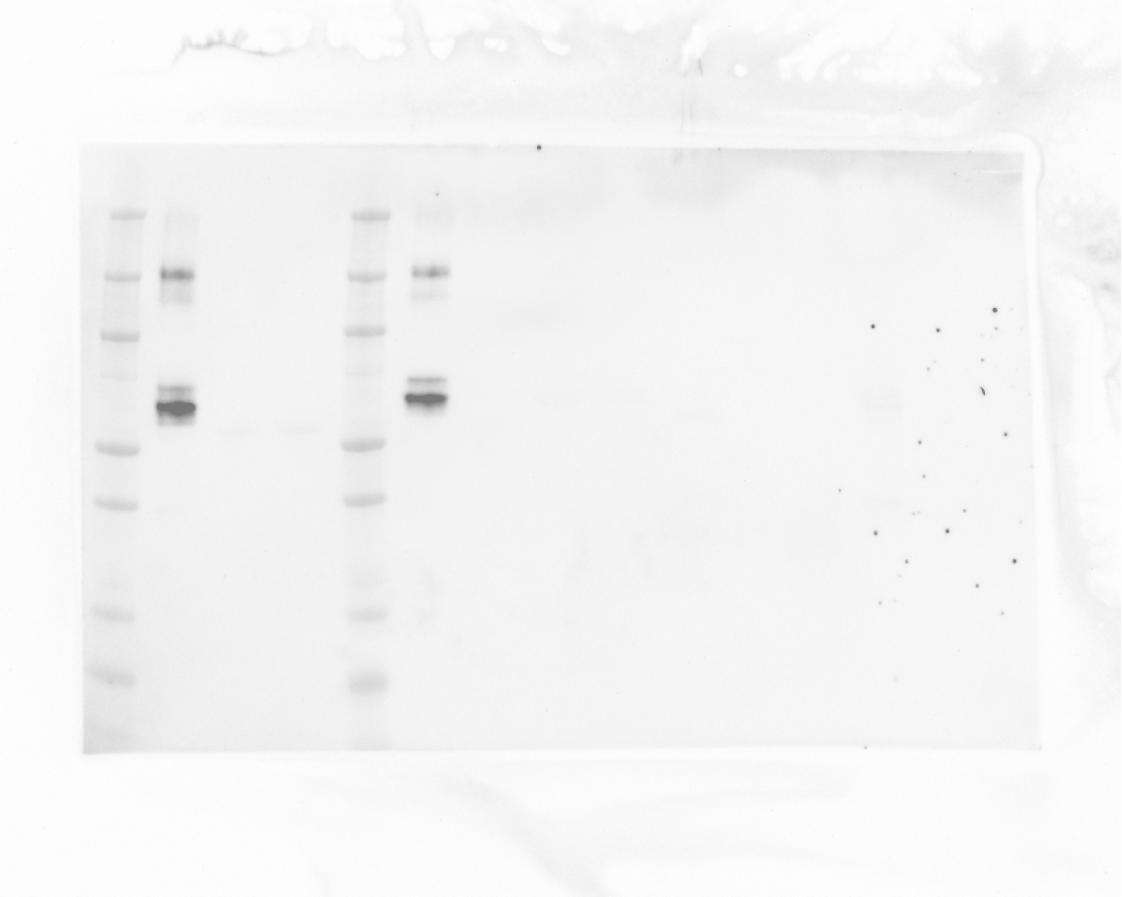

Supplement: Figure 3—source data 9. [file elife-95118-fig3-data9.zip › Figure 3-source data 9/Figure3BC_anti-PANX1-pY308.tif]

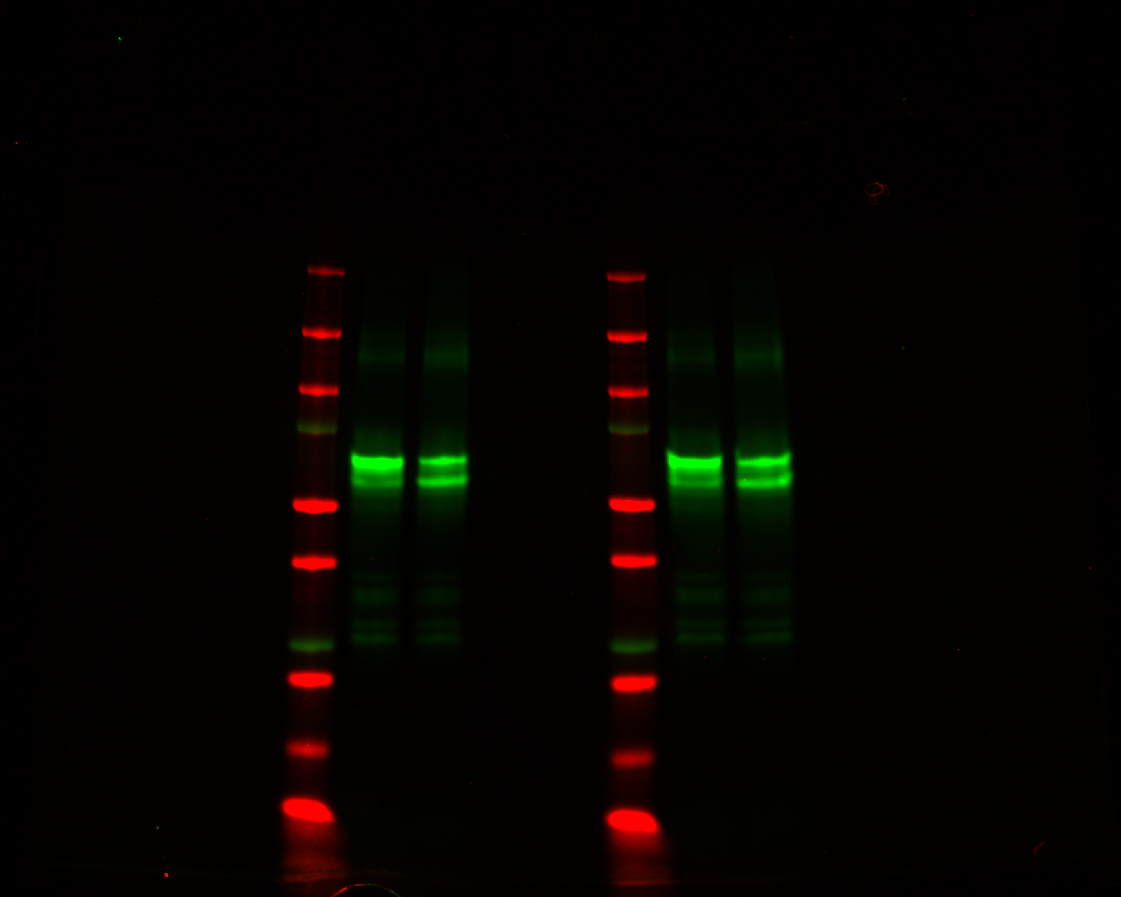

Supplement: Figure 3—source data 10. [file elife-95118-fig3-data10.zip › Figure 3-source data 10/Figure3D_fluorescence.tif]

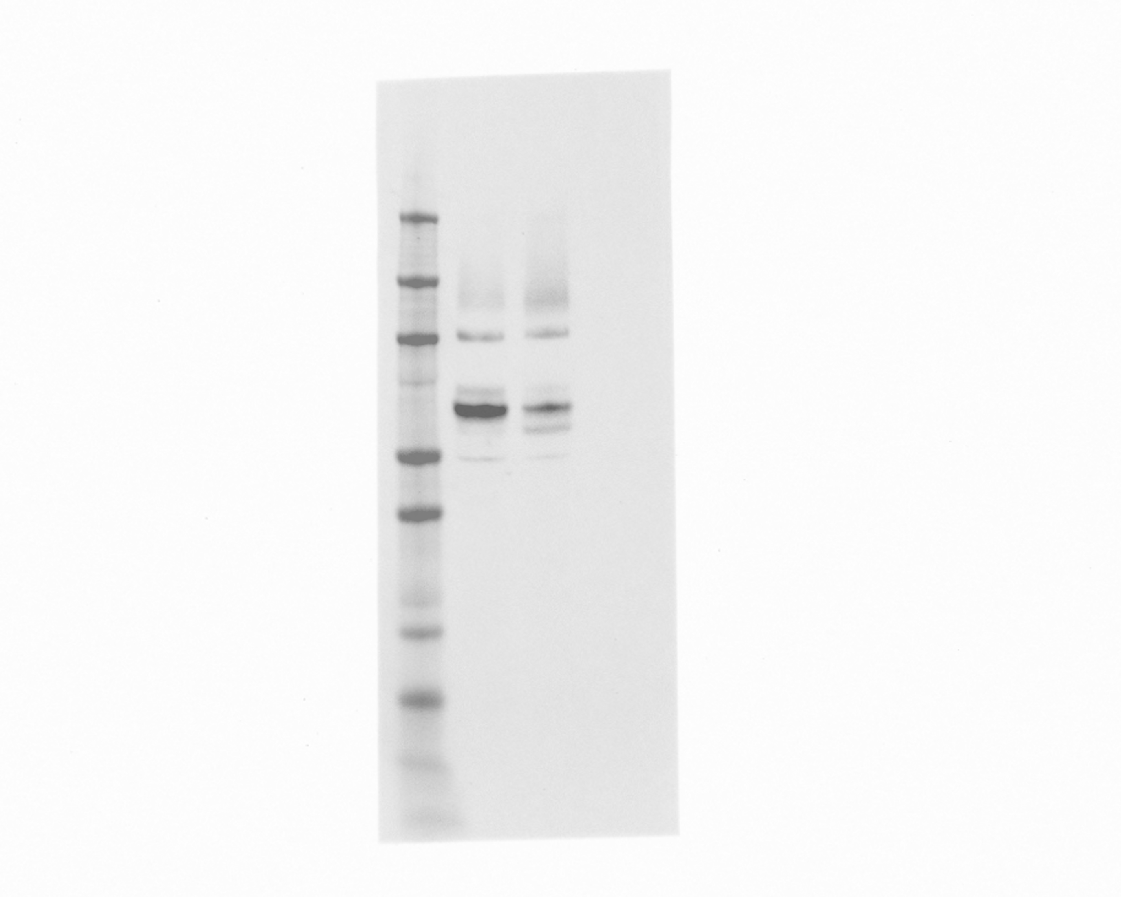

Supplement: Figure 3—source data 11. [file elife-95118-fig3-data11.zip › Figure 3-source data 11/Figure3D_anti-PANX1.tif]

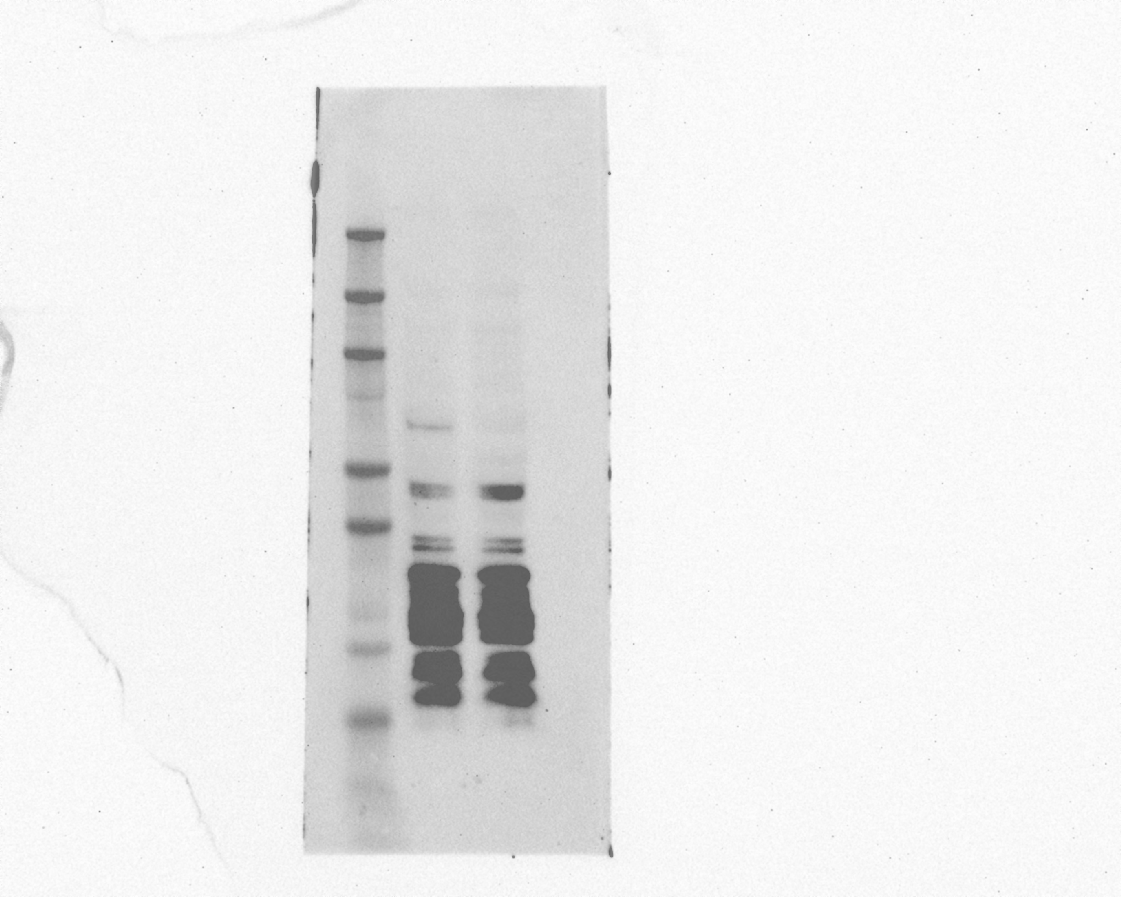

Supplement: Figure 3—source data 12. [file elife-95118-fig3-data12.zip › Figure 3-source data 12/Figure3D_anti-PANX1-pY308.tif]

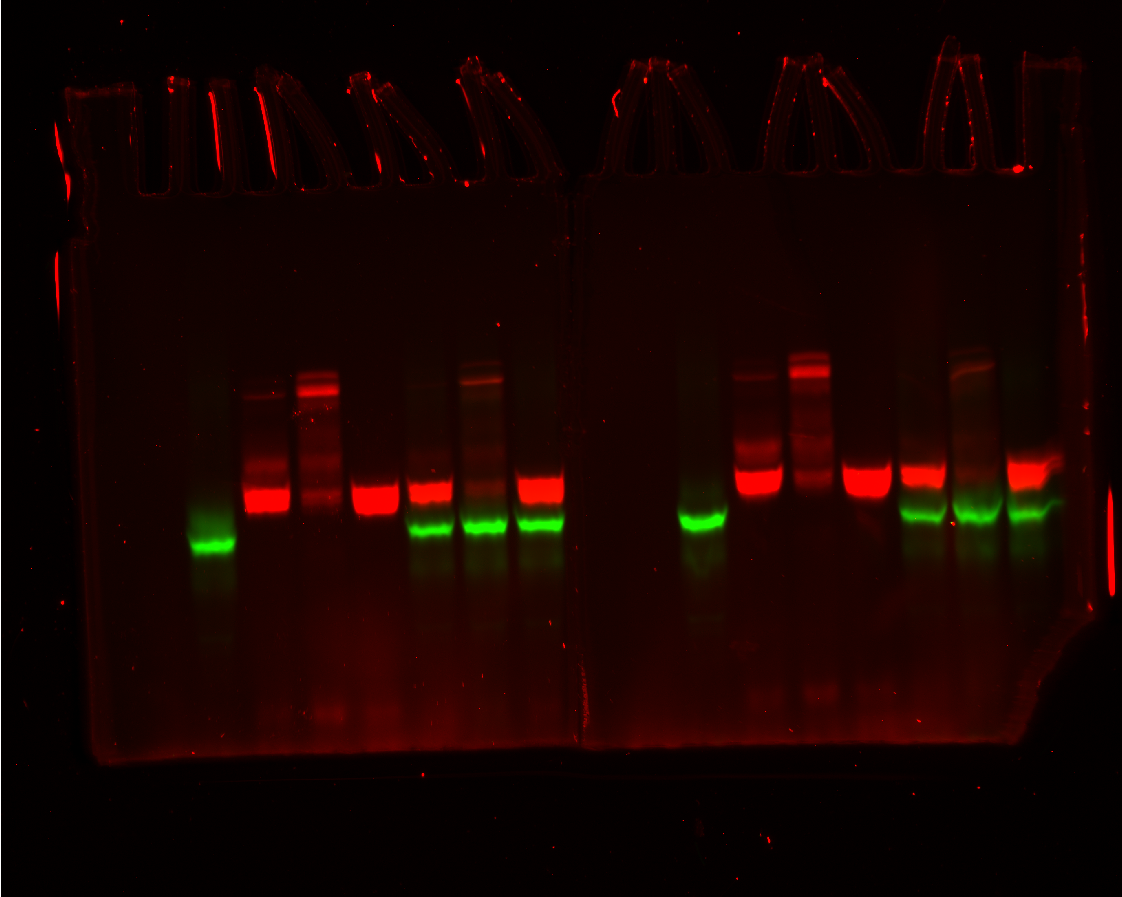

Supplement: Figure 4—source data 2. [file elife-95118-fig4-data2.zip › Figure 4 source data 2/Figure4A_fluorescence.tif]

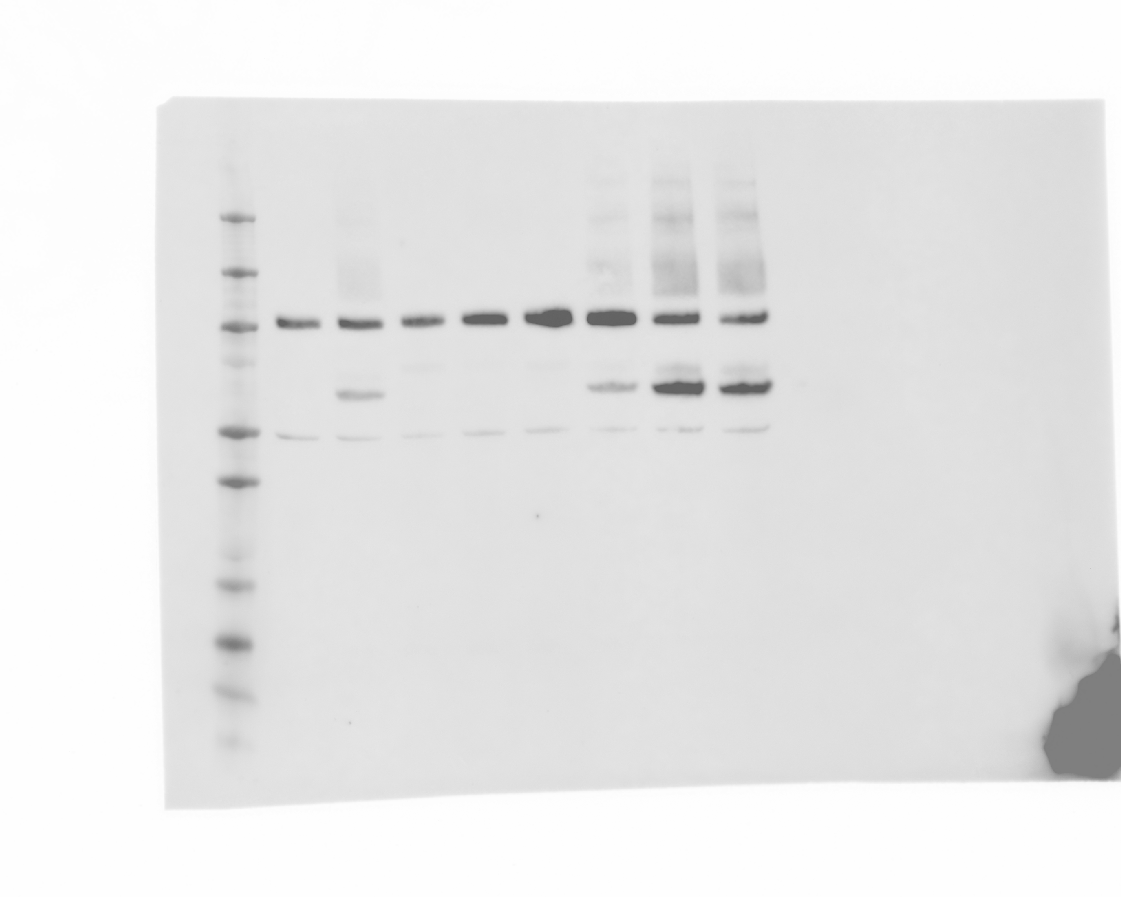

Supplement: Figure 4—source data 3. [file elife-95118-fig4-data3.zip › Figure 4 source data 3/Figure4B_anti-PANX1.tif]

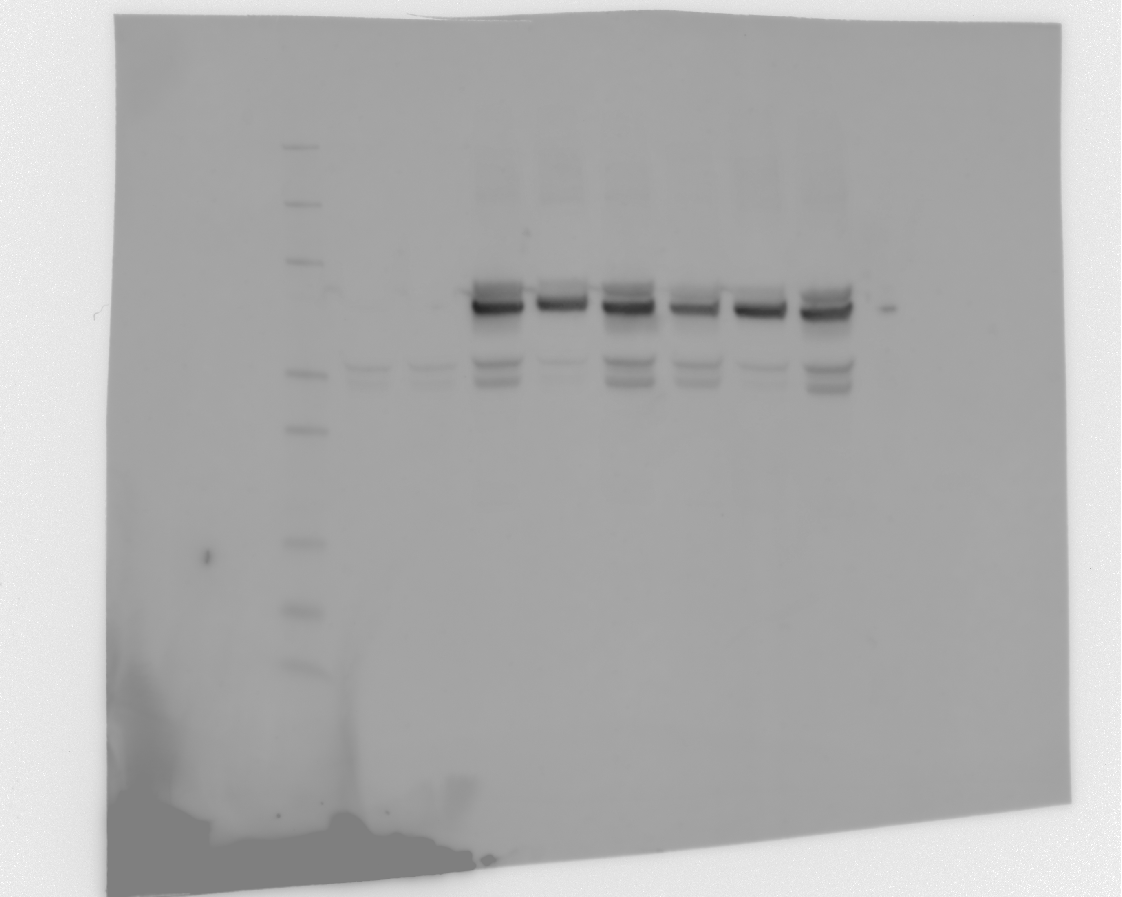

Supplement: Figure 4—source data 4. [file elife-95118-fig4-data4.zip › Figure 4 source data 4/Figure4B_anti-Src.tif]

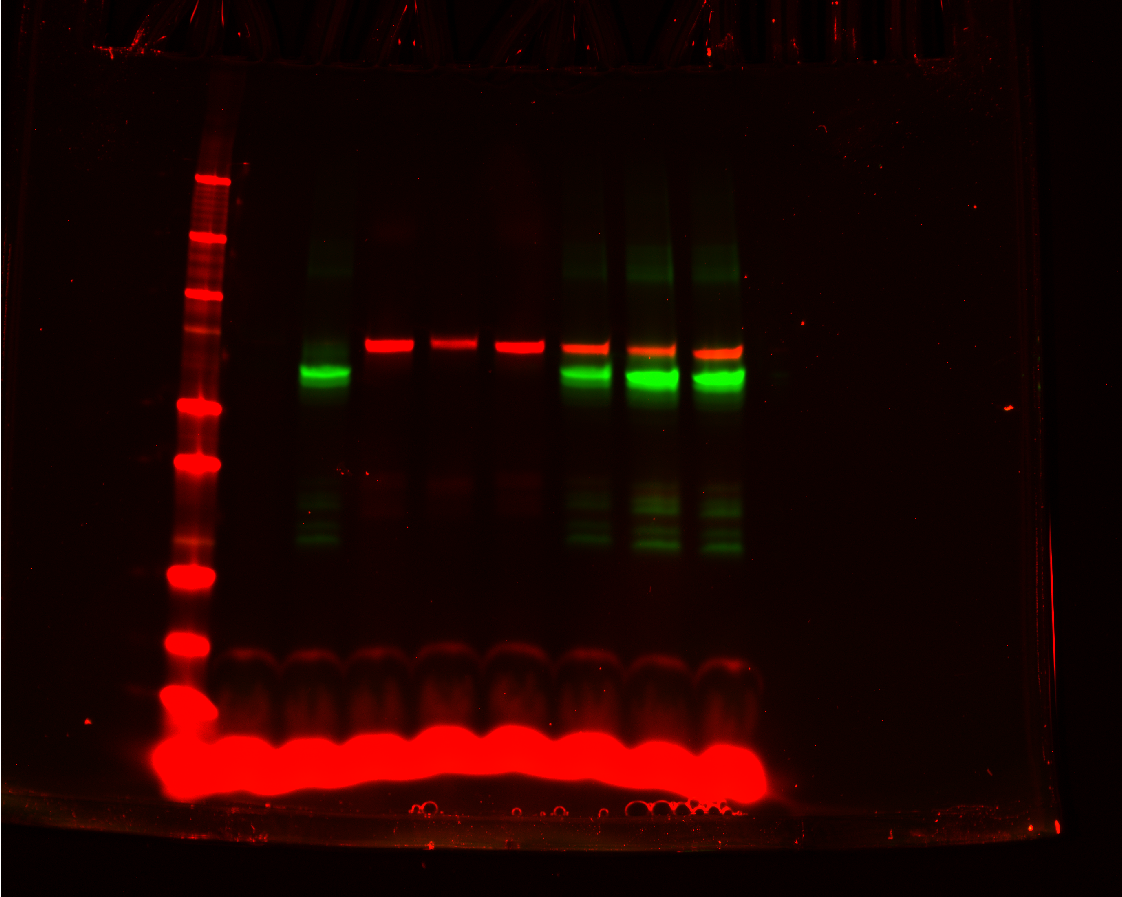

Supplement: Figure 4—source data 5. [file elife-95118-fig4-data5.zip › Figure 4 source data 5/Figure4B_fluorescence.tif]

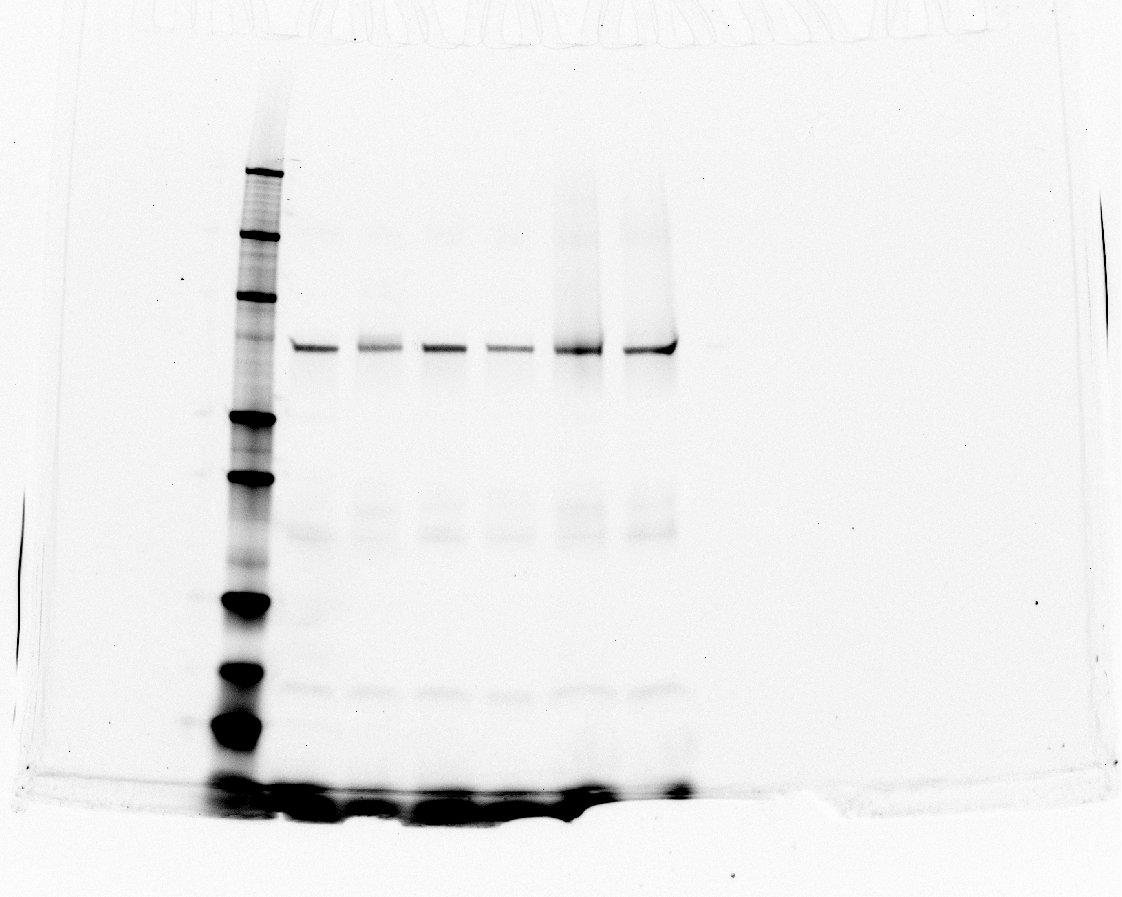

Supplement: Figure 4—figure supplement 1—source data 2. [file elife-95118-fig4-figsupp1-data2.zip › Figure 4 figure supplement 1 source data 2/Figure4_supp1_SDSPAGE.tif]

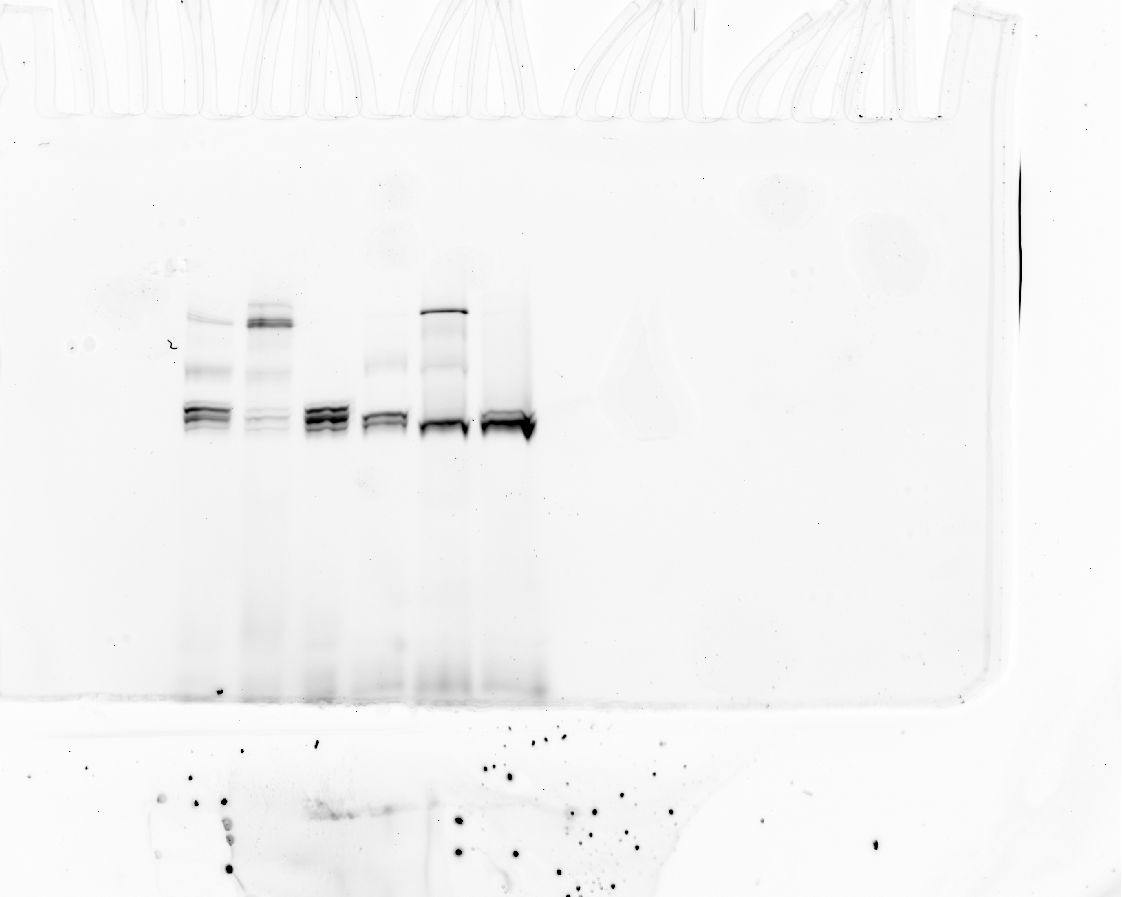

Supplement: Figure 4—figure supplement 1—source data 3. [file elife-95118-fig4-figsupp1-data3.zip › Figure 4 figure supplement 1 source data 3/Figure4_supp1_PhosTag.tif]

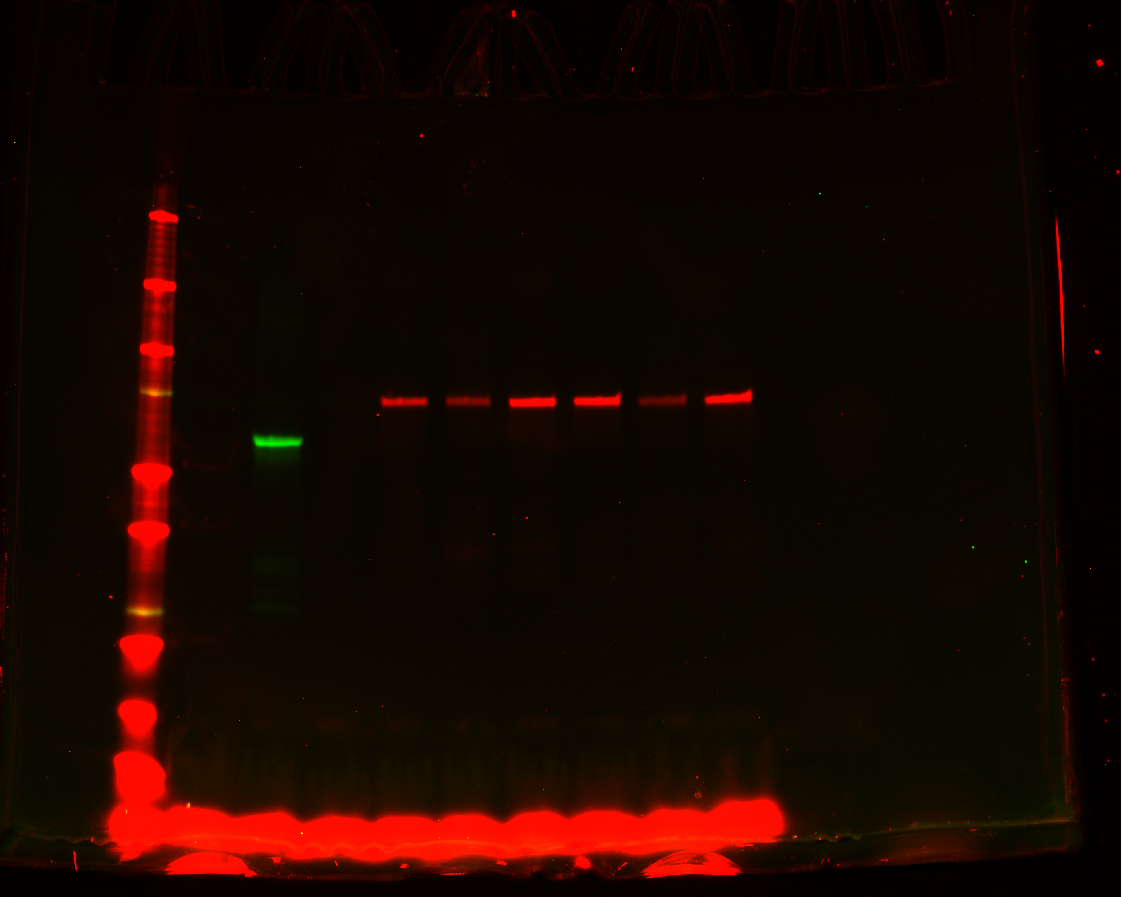

Supplement: Figure 4—figure supplement 2—source data 2. [file elife-95118-fig4-figsupp2-data2.zip › figure 4 figure supplement 2 source data 2/figure4_suppl2_fluorescence.tif]

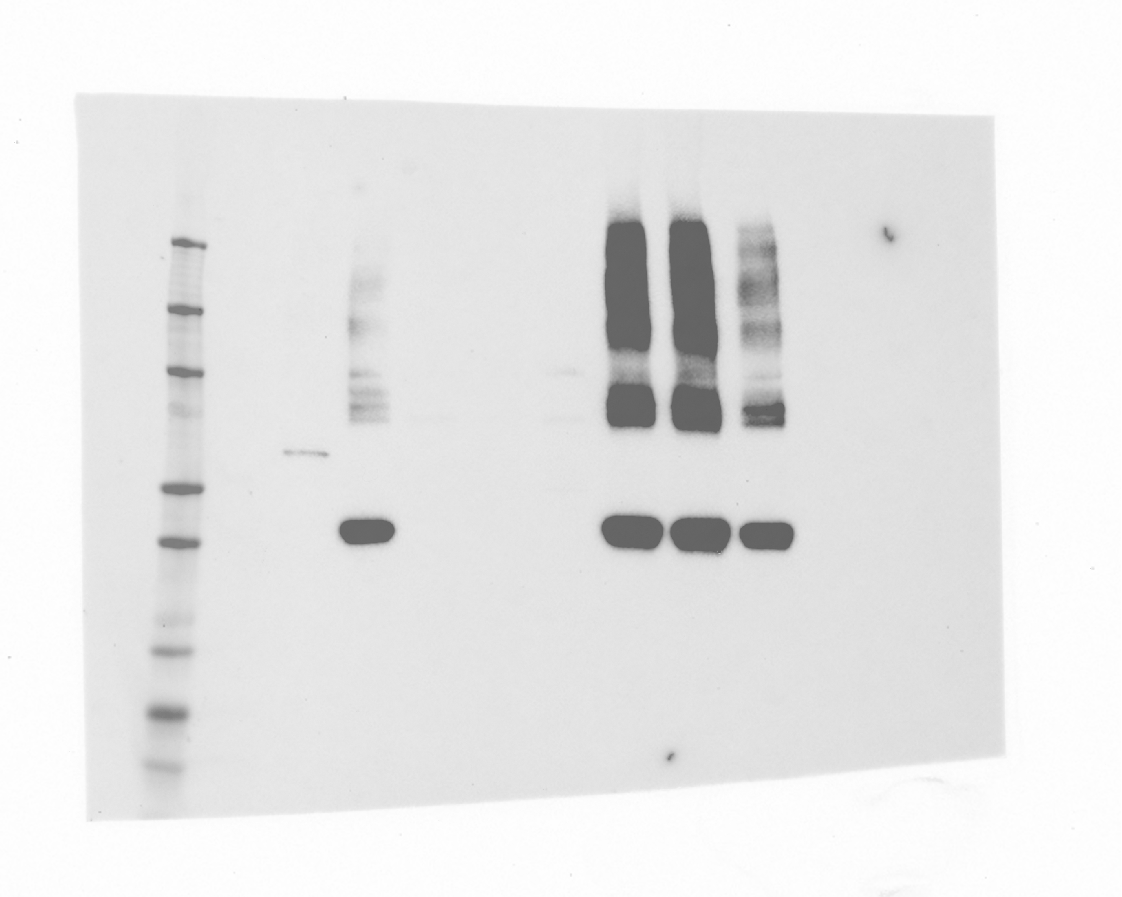

Supplement: Figure 4—figure supplement 2—source data 3. [file elife-95118-fig4-figsupp2-data3.zip › figure 4 figure supplement 2 source data 3/figure4_suppl2_SDSPAGE_anti-PANX1.tif]

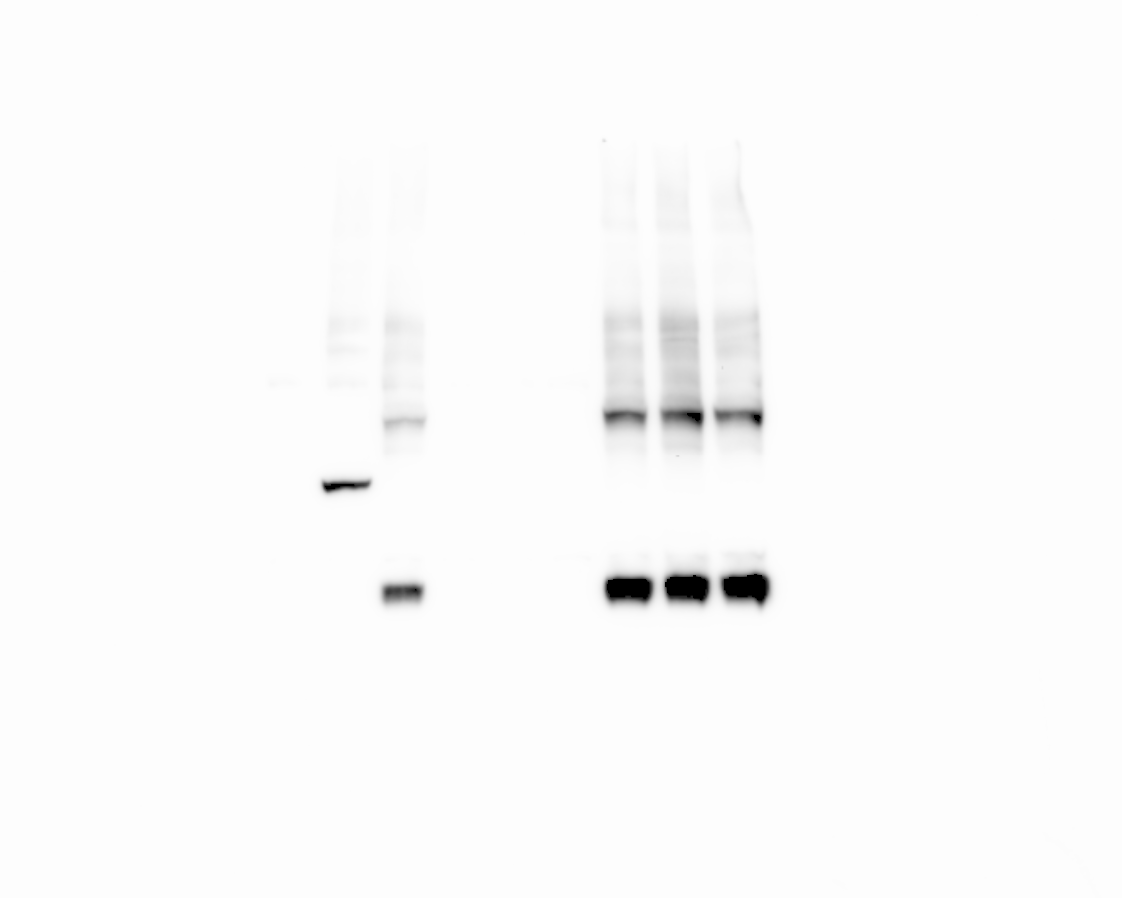

Supplement: Figure 4—figure supplement 2—source data 4. [file elife-95118-fig4-figsupp2-data4.zip › figure 4 figure supplement 2 source data 4/figure4_suppl2_PhosTag_anti-PANX1.tif]

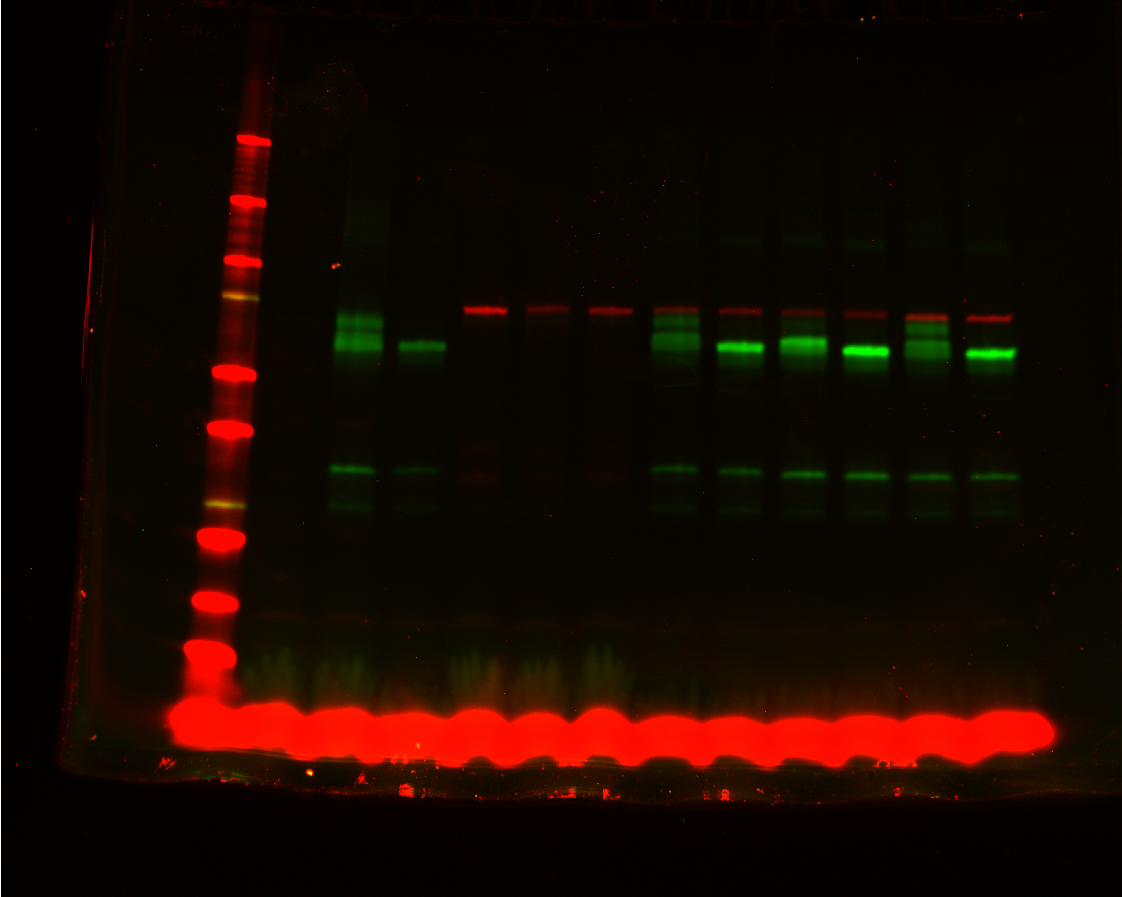

Supplement: Figure 5—source data 2. [file elife-95118-fig5-data2.zip › Figure 5 source data 2/Figure5A.tif]

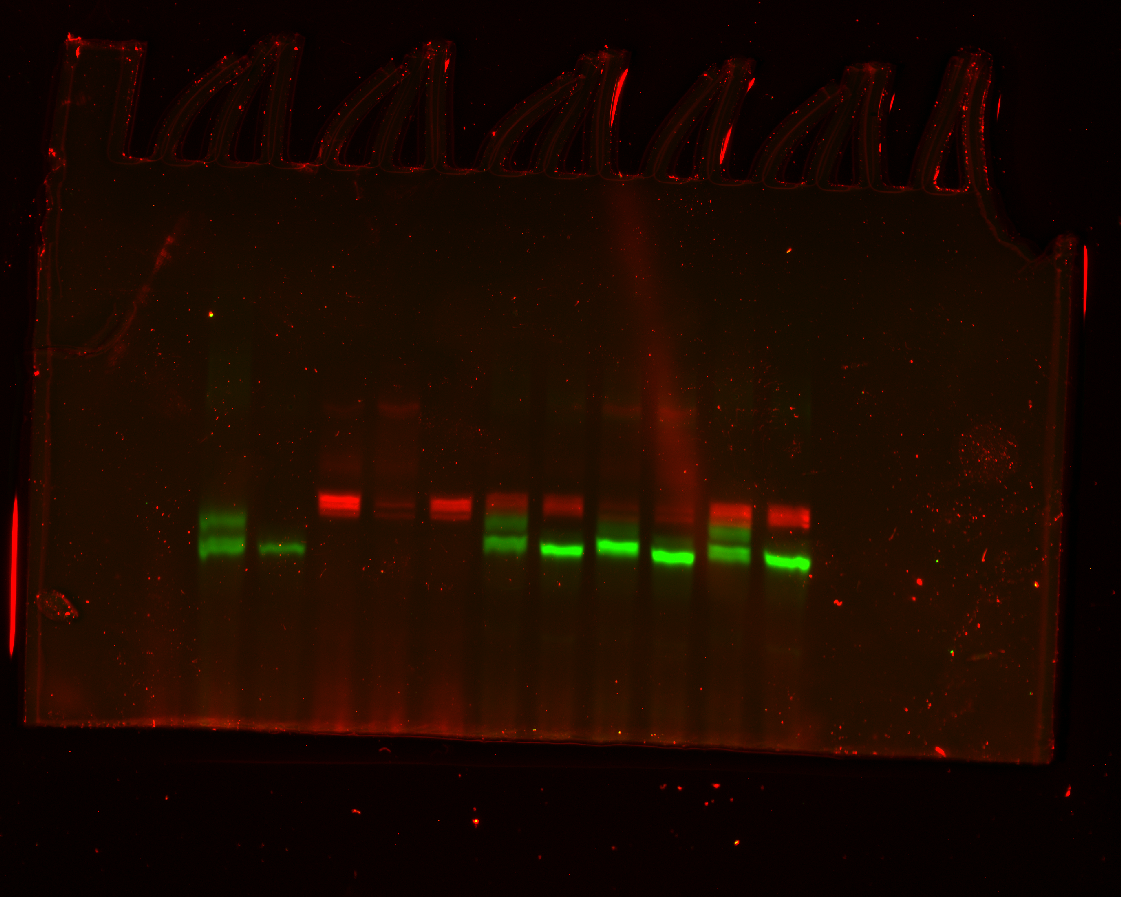

Supplement: Figure 5—source data 3. [file elife-95118-fig5-data3.zip › Figure 5 source data 3/Figure5B.tif]

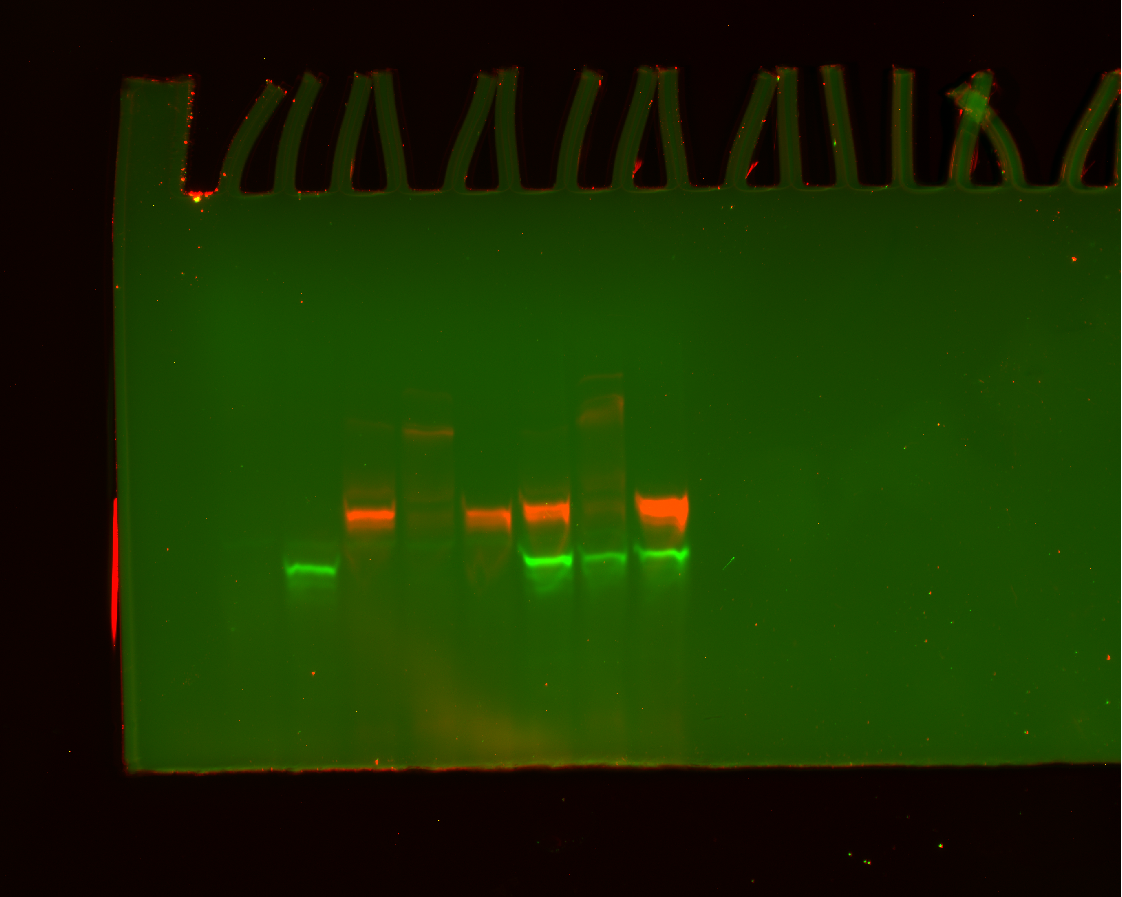

Supplement: Figure 6—source data 2. [file elife-95118-fig6-data2.zip › Figure 6 source data 2/Figure6A.tif]

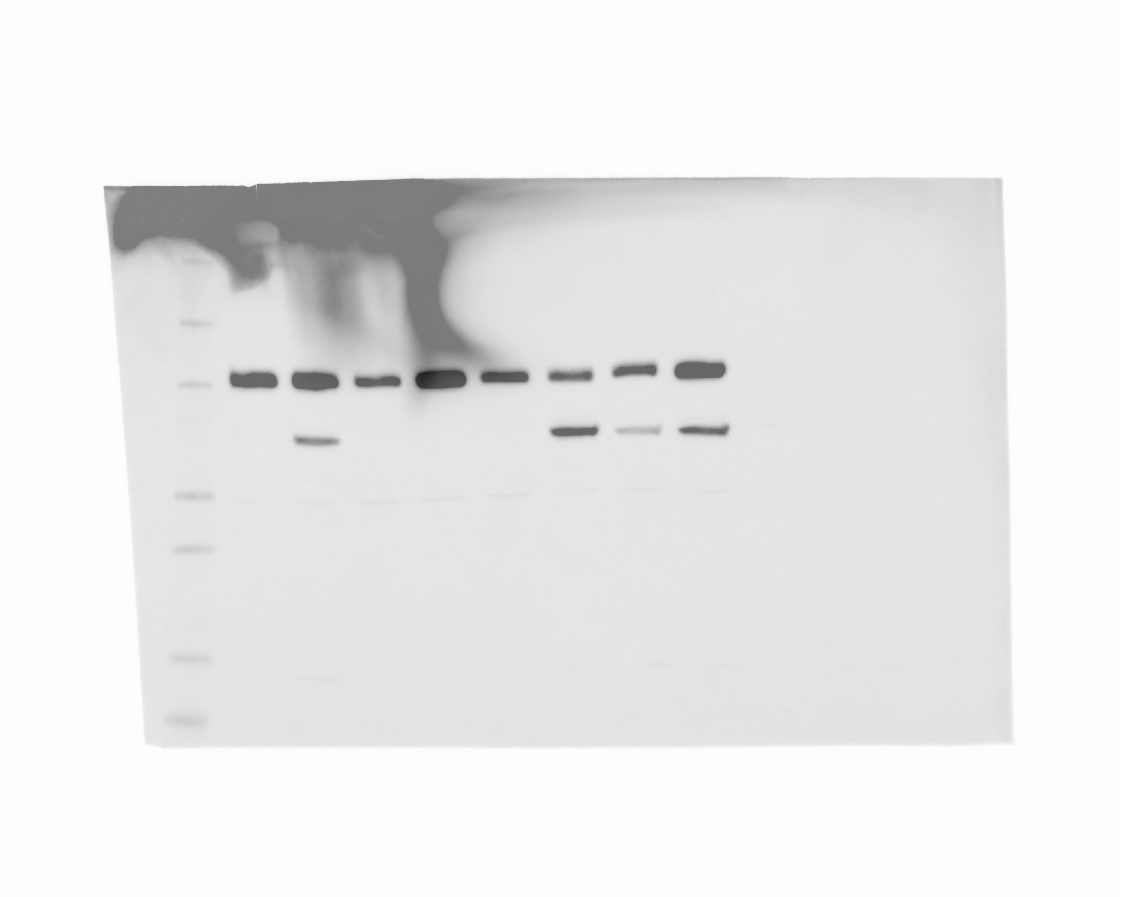

Supplement: Figure 6—source data 3. [file elife-95118-fig6-data3.zip › Figure 6 source data 3/Figure6B_anti-PANX1.tif]

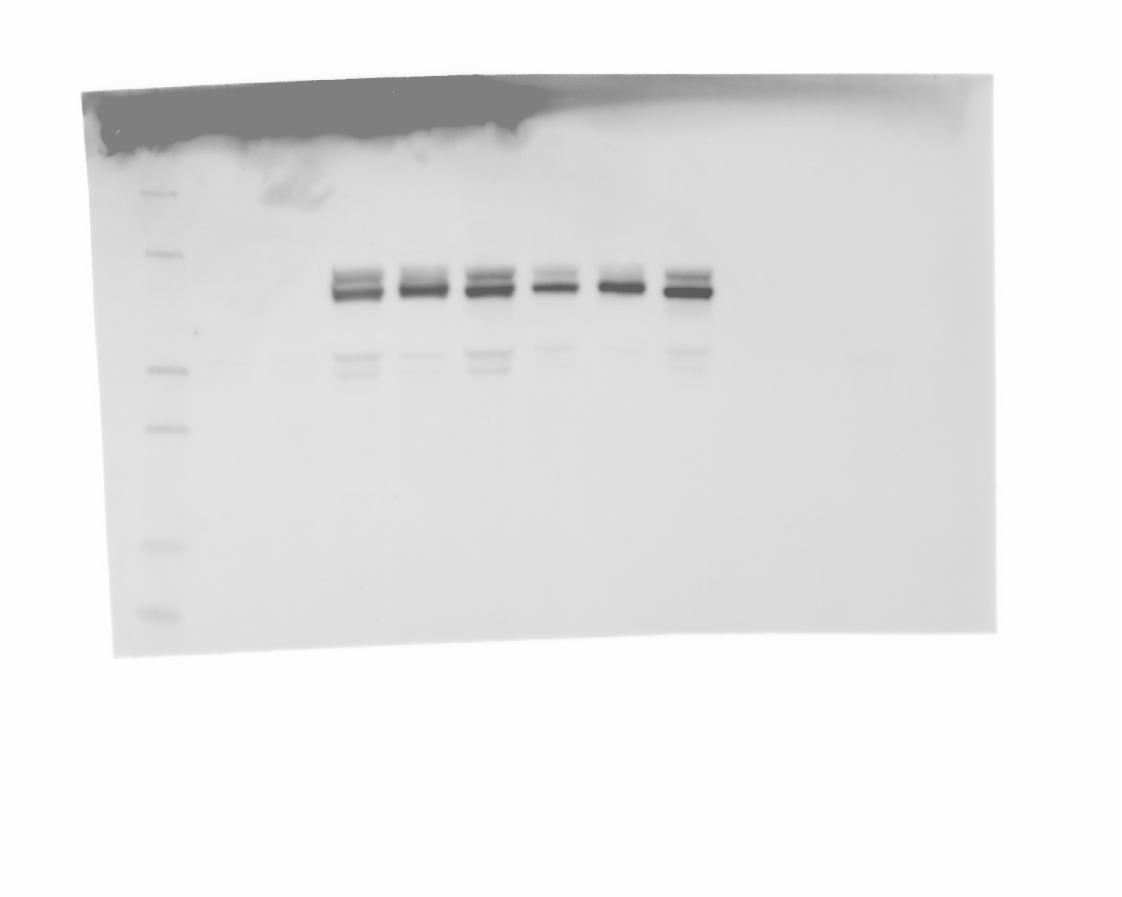

Supplement: Figure 6—source data 4. [file elife-95118-fig6-data4.zip › Figure 6 source data 4/Figure6B_anti-Src.tif]

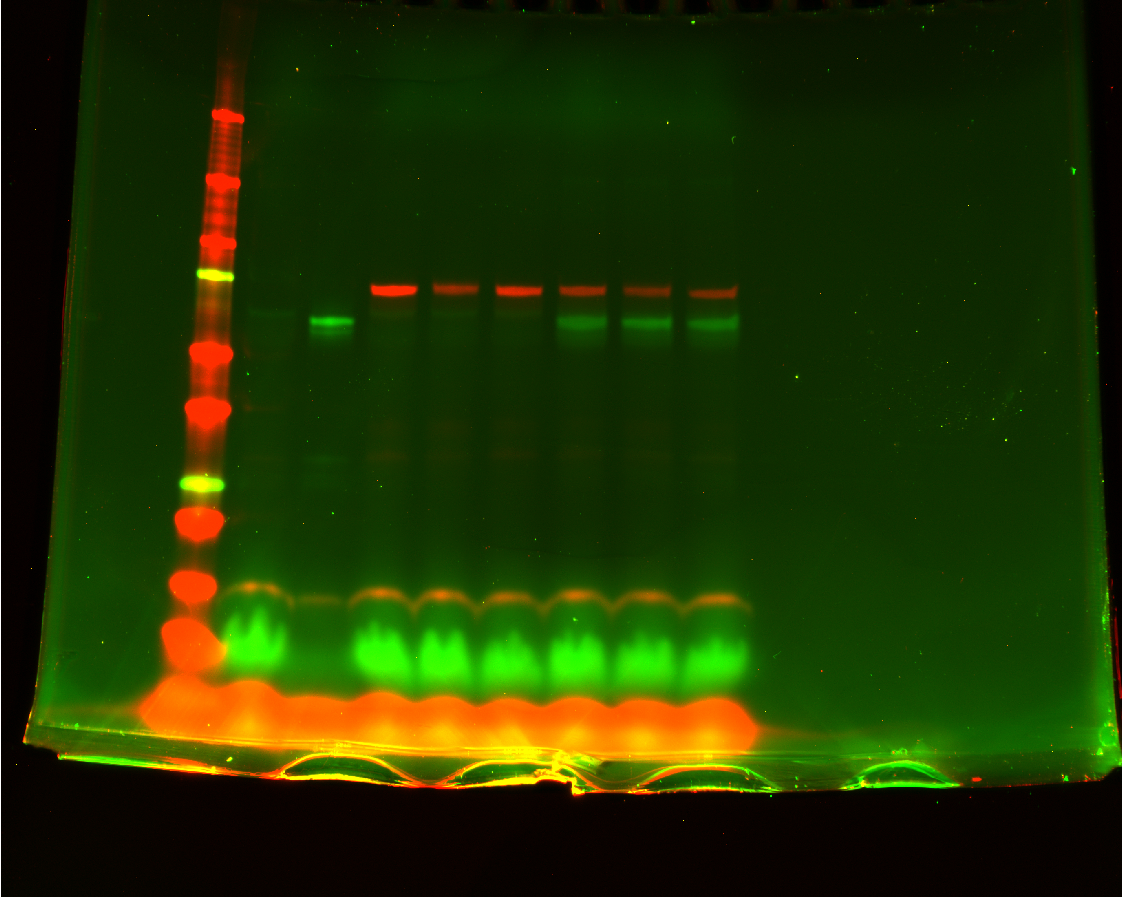

Supplement: Figure 6—source data 5. [file elife-95118-fig6-data5.zip › Figure 6 source data 5/Figure6B_fluorescence.tif]

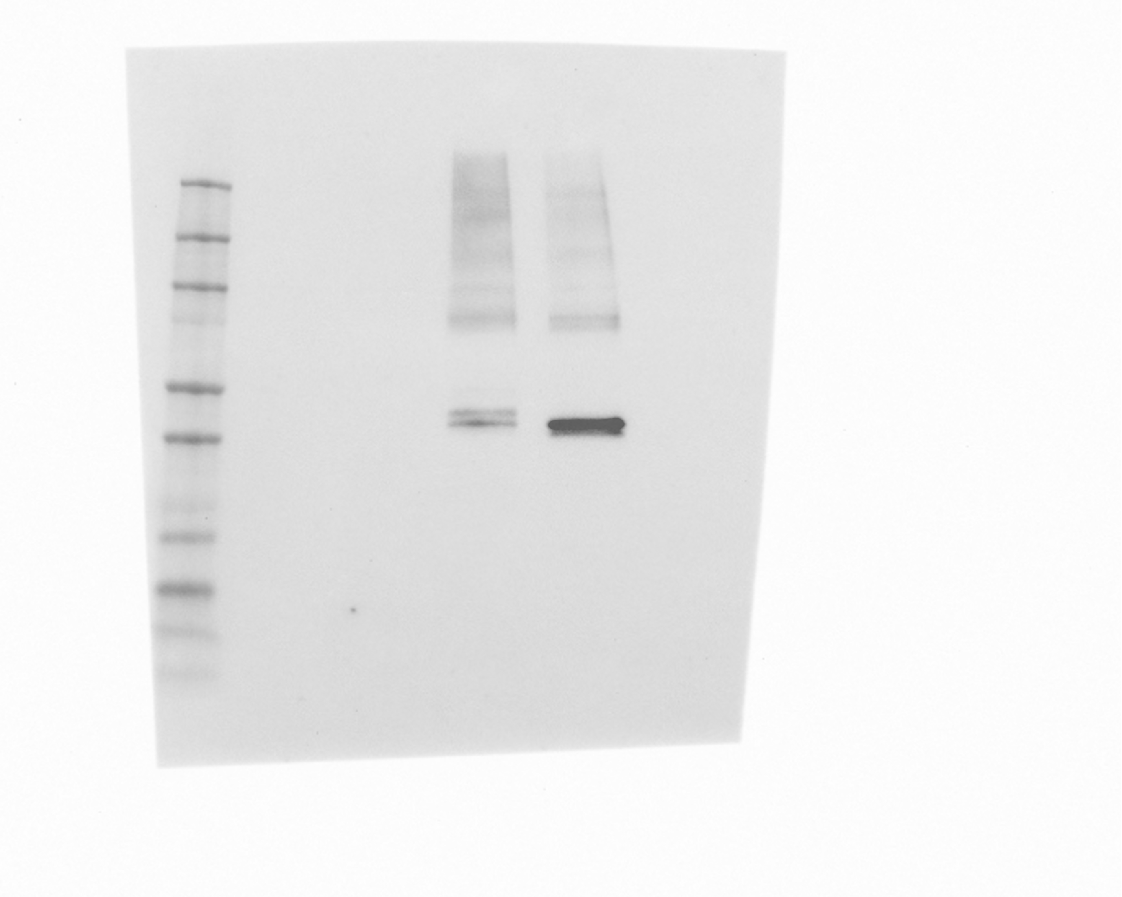

Supplement: Figure 6—figure supplement 1—source data 2. [file elife-95118-fig6-figsupp1-data2.zip › figure 6 figure supplement 1 source data 2/figure6_suppl1_left_panel.tif]

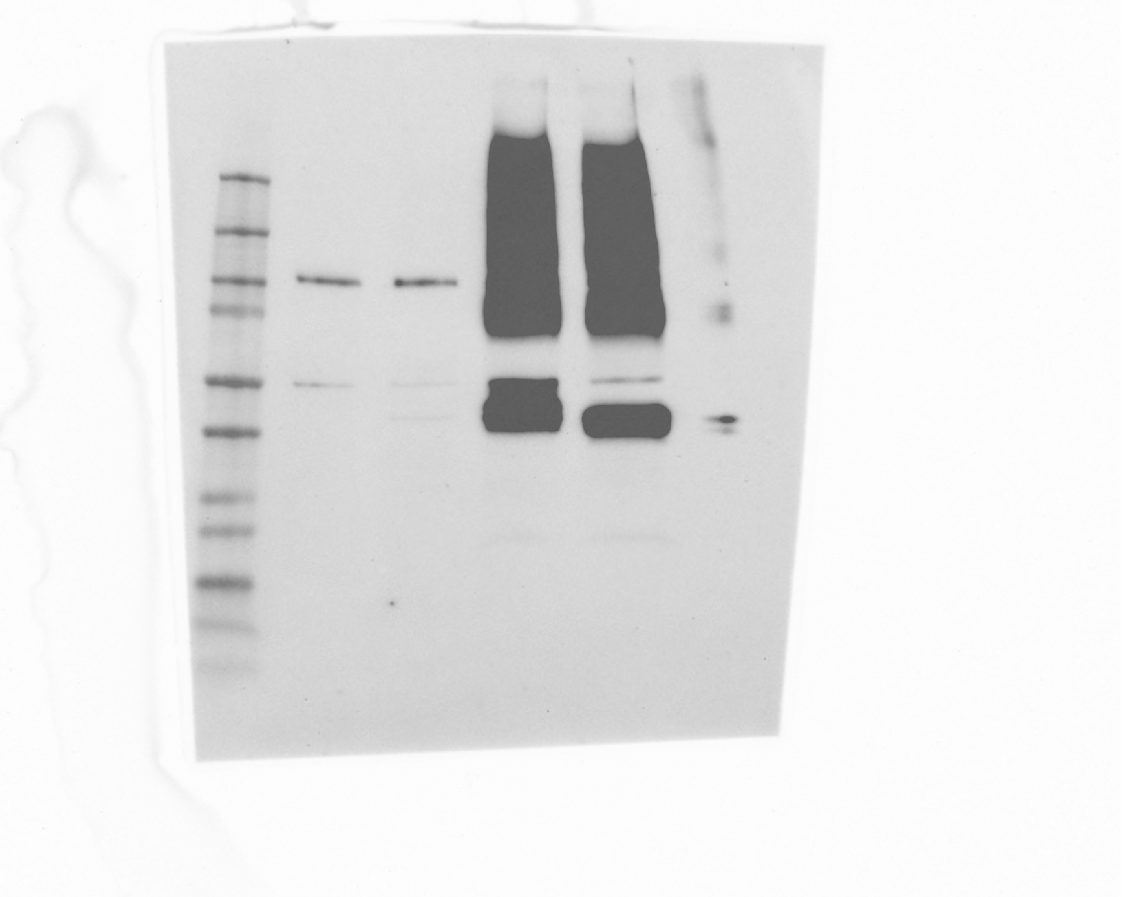

Supplement: Figure 6—figure supplement 1—source data 3. [file elife-95118-fig6-figsupp1-data3.zip › figure 6 figure supplement 1 source data 3/figure6_suppl1_right_panel.tif]

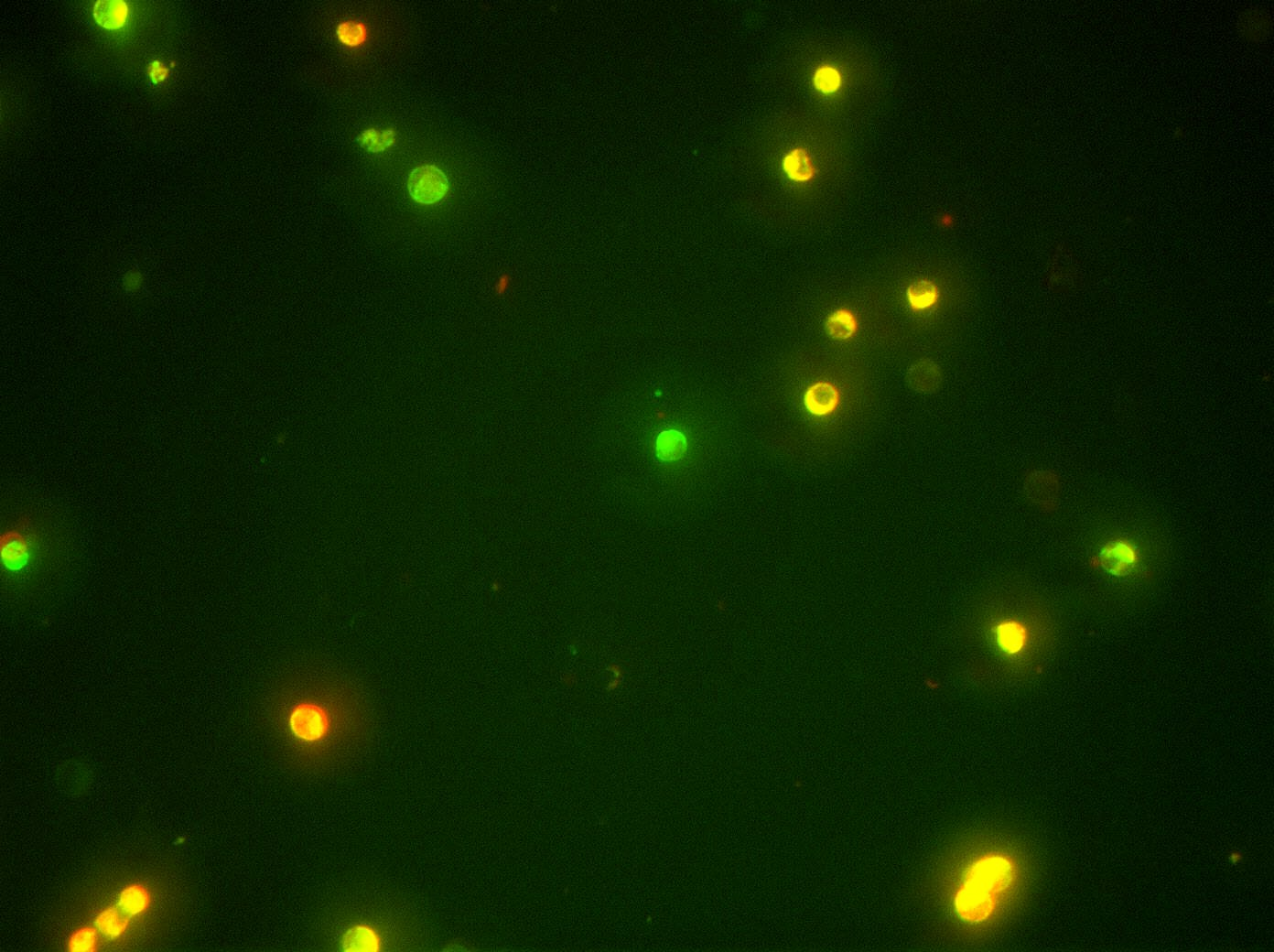

Supplement: Figure 7—source data 1. [file elife-95118-fig7-data1.zip › figure 7-source data 1/GFP_mCherry_merge.tif]

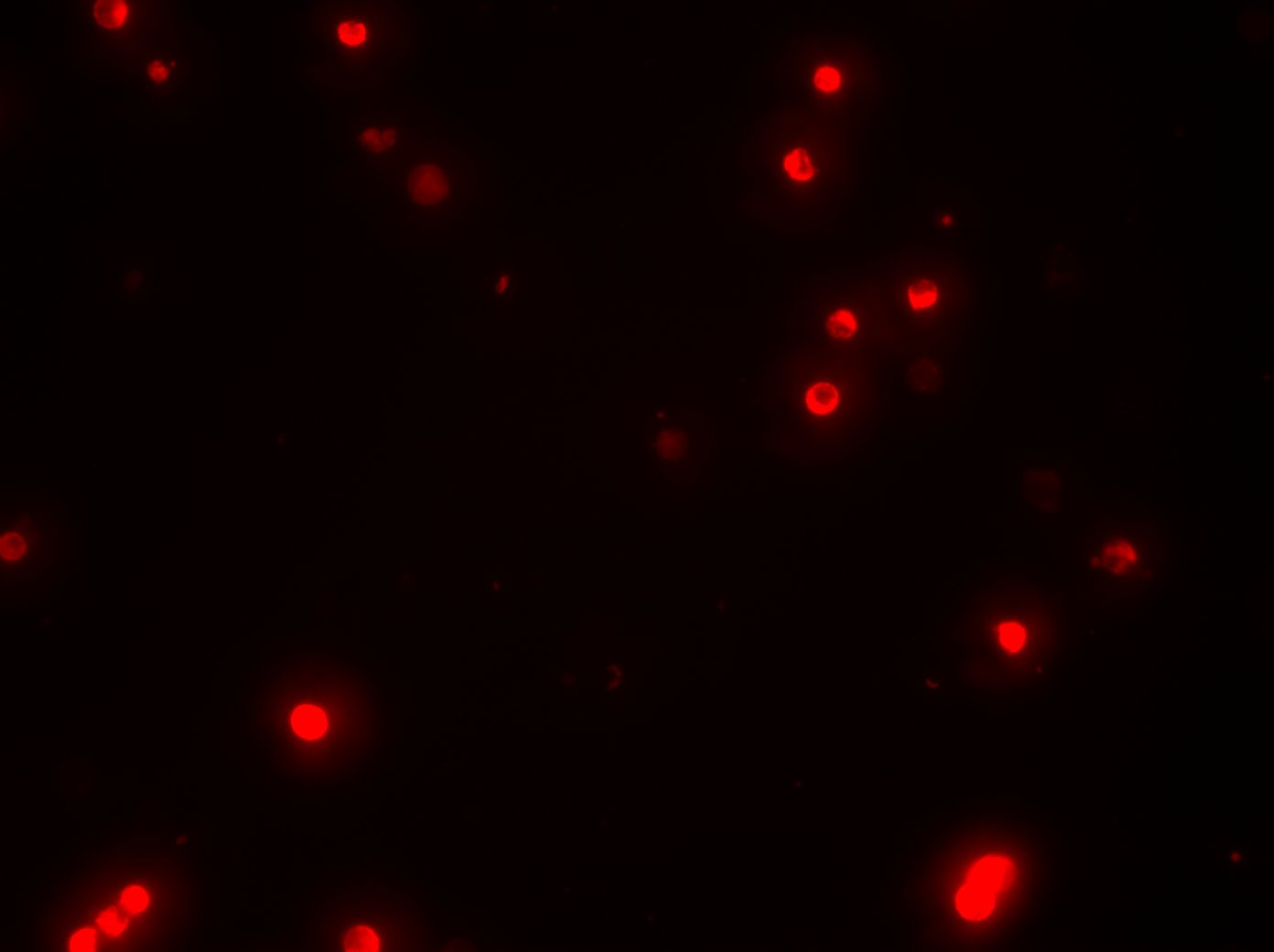

Supplement: Figure 7—source data 1. [file elife-95118-fig7-data1.zip › figure 7-source data 1/mCherry.tif]

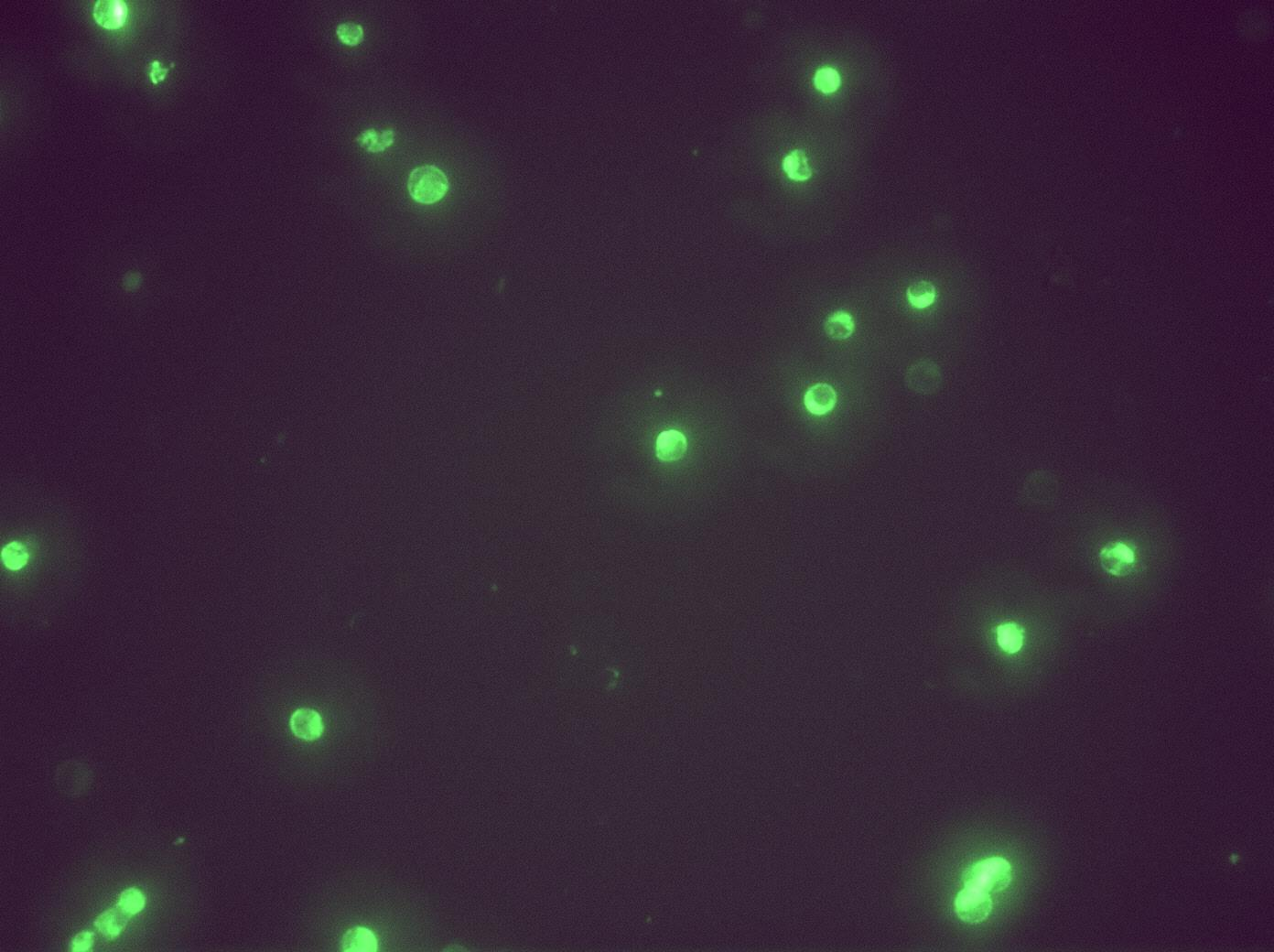

Supplement: Figure 7—source data 1. [file elife-95118-fig7-data1.zip › figure 7-source data 1/GFP.tif]
