# Supplementary material for: Human pannexin 1 channel is not phosphorylated by Src tyrosine kinase at Tyr199 and Tyr309
Source: eLife. 2024 May 23;13:RP95118. doi: 10.7554/eLife.95118 (PMC11115448; doi:10.7554/eLife.95118)
Supplement: Figure 3—source data 1. [file elife-95118-fig3-data1.zip › Figure 3-source data 1/figure3_source_data_1.pdf]

Figure 3-source data 1

First panel of Figure 3A

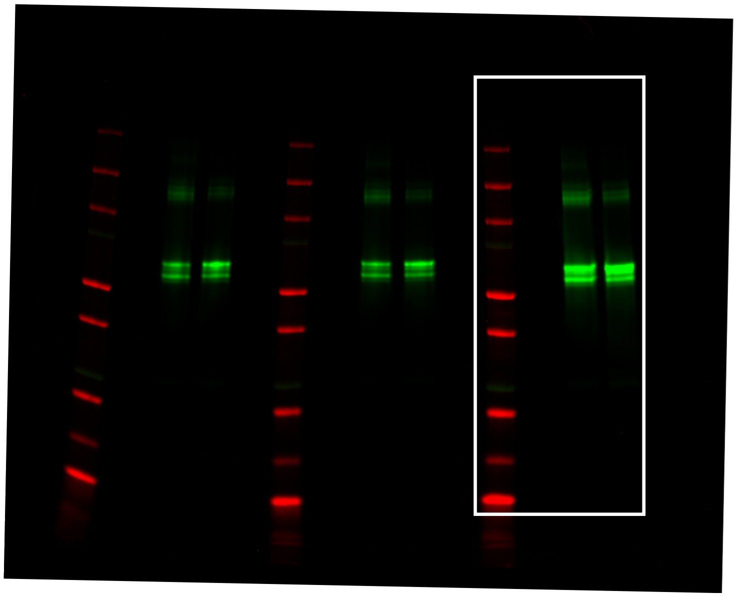

In-gel fluorescence

Second panel of Figure 3A

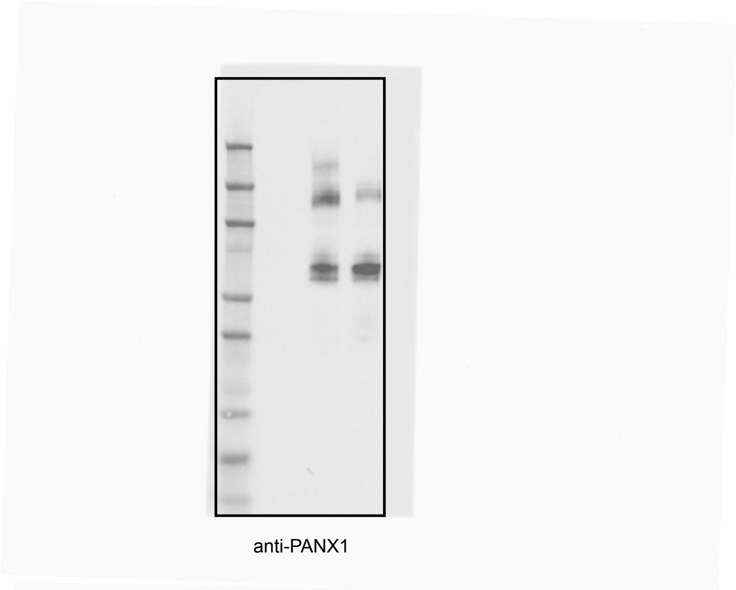

anti-PANX1

Third panel of Figure 3A

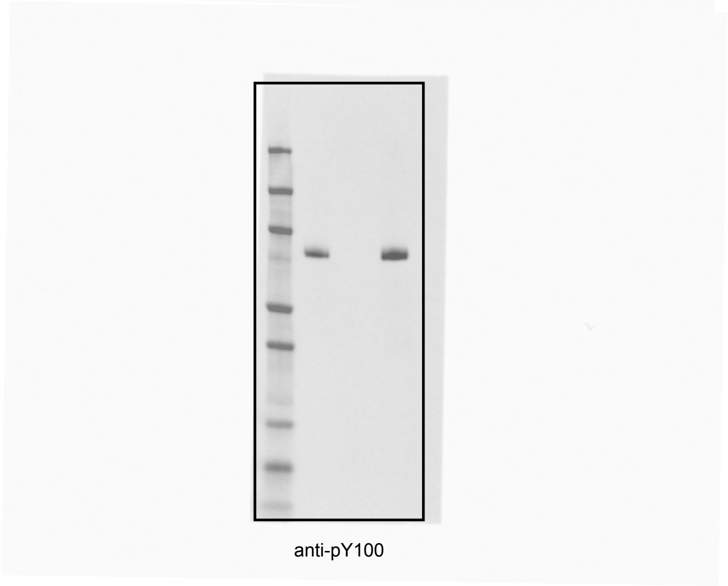

anti-pY100

Fourth panel of Figure 3A

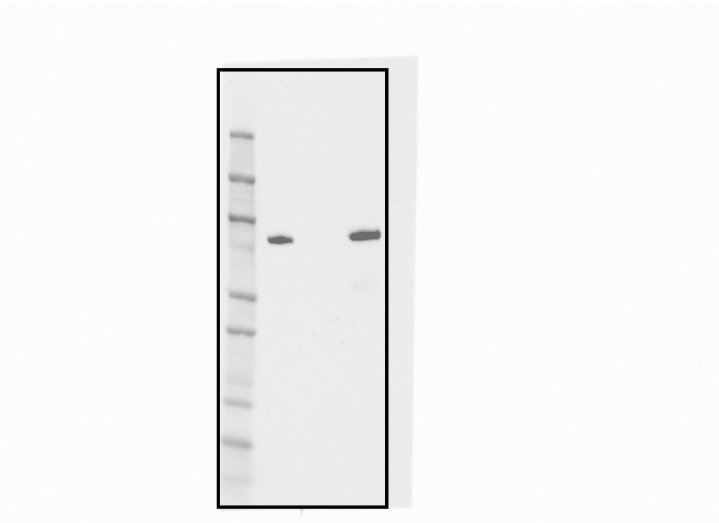

anti-Src

Fifth panel of Figure 3A

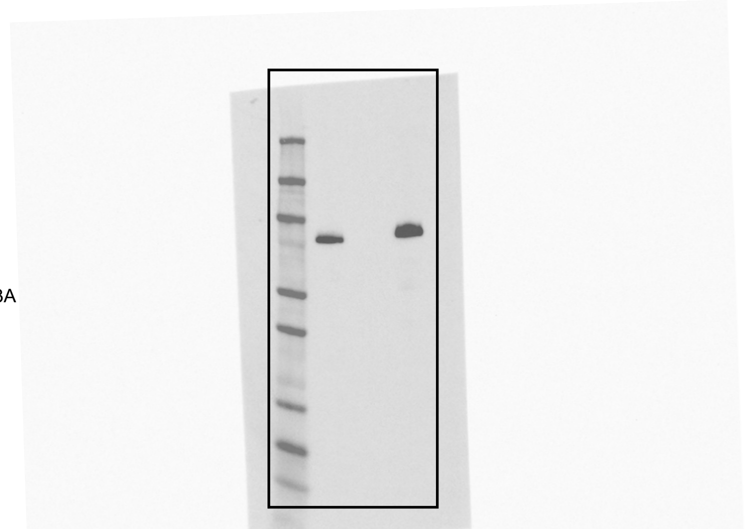

anti-PANX1-pY198

Sixth panel of Figure 3A

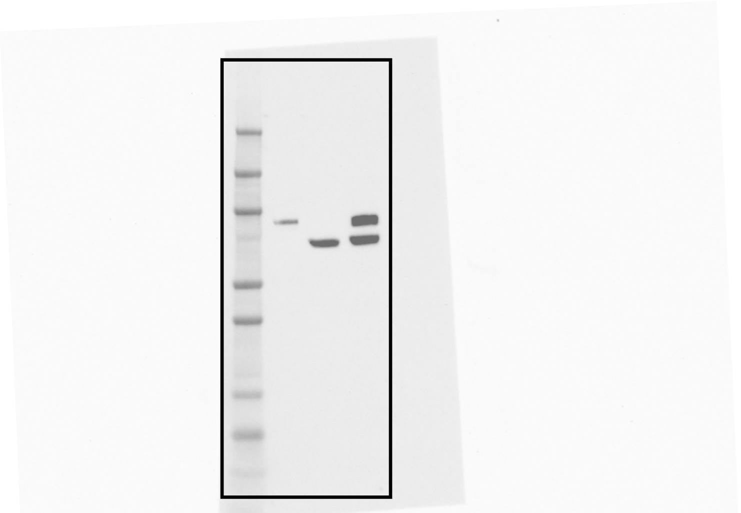

anti-PANX1-pY308

Left panel of Figure 3B

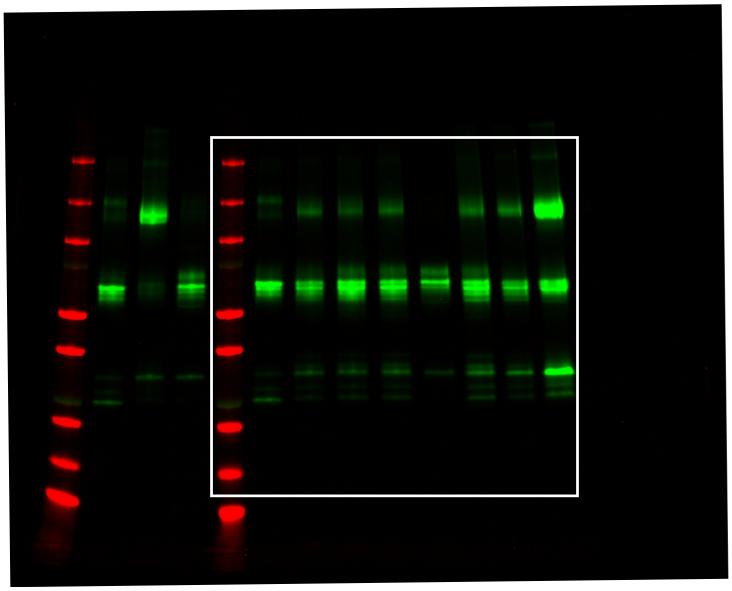

In-gel fluorescence

Right panel of Figure 3B

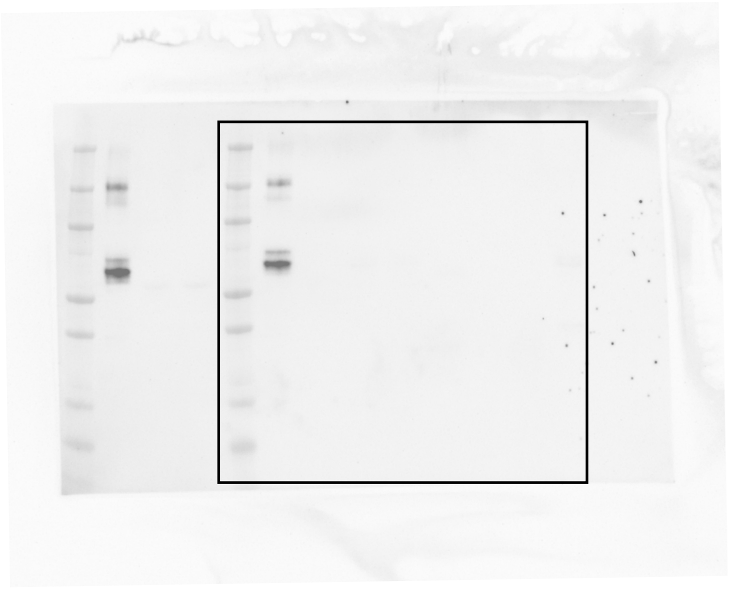

anti-PANX1-pY308

Left panel of Figure 3C

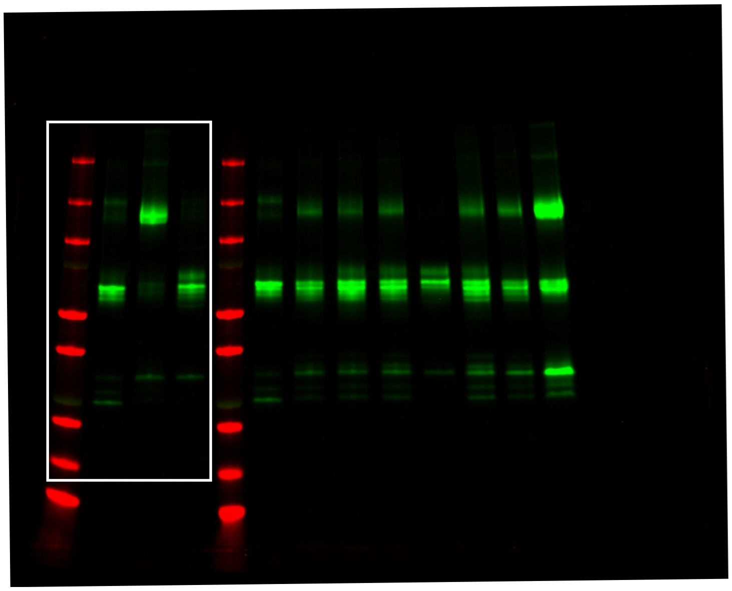

In-gel fluorescence

Right panel of Figure 3C

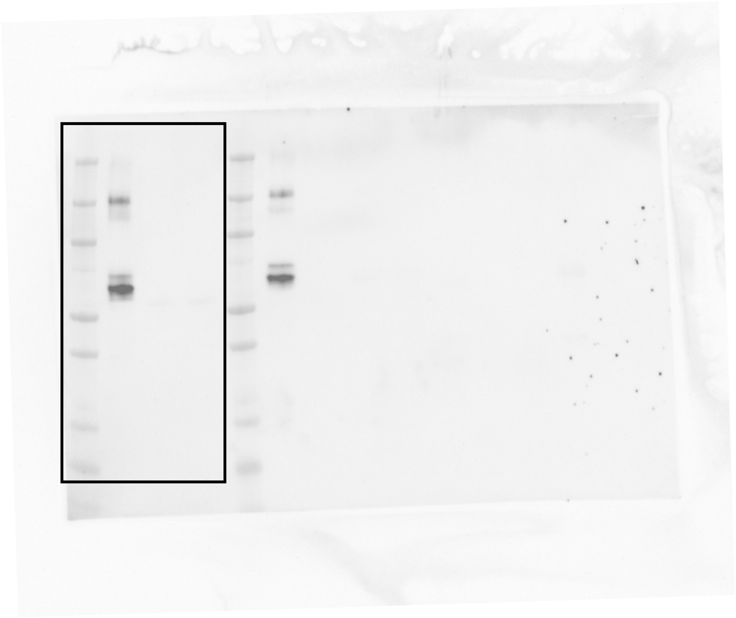

anti-PANX1-pY308

Left panel of Figure 3D

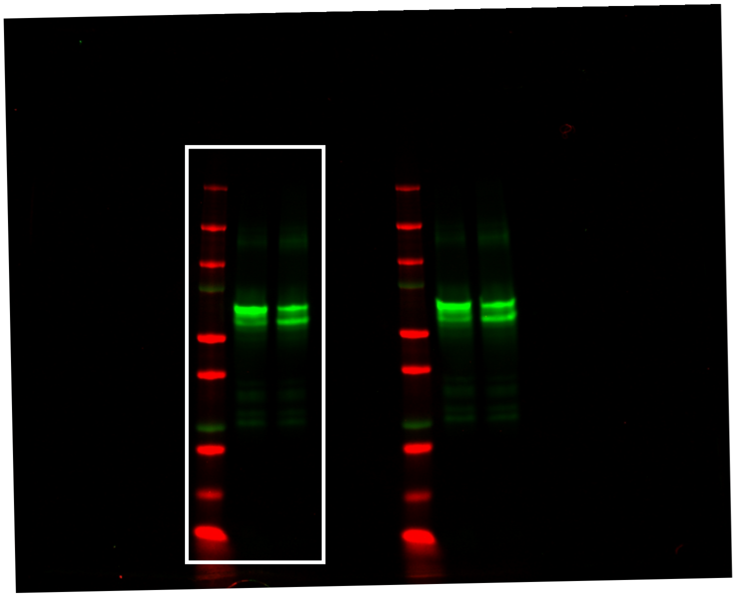

In-gel fluorescence

Middle panel of Figure 3D

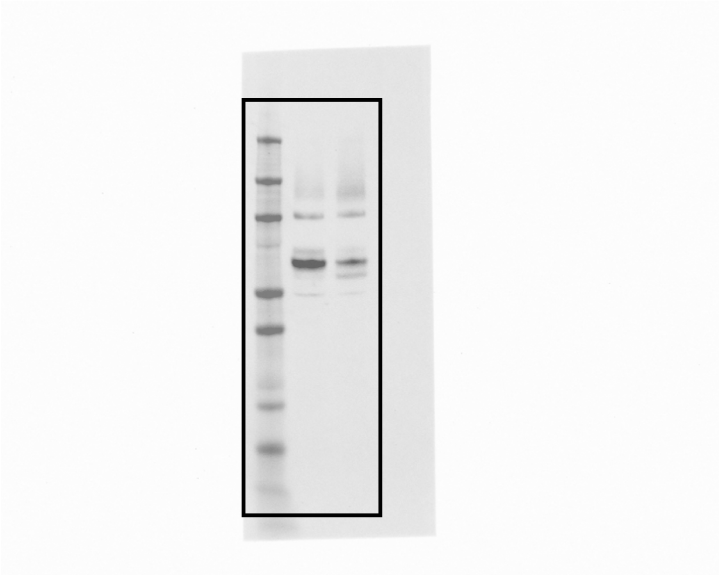

anti-PANX1

Right panel of Figure 3D

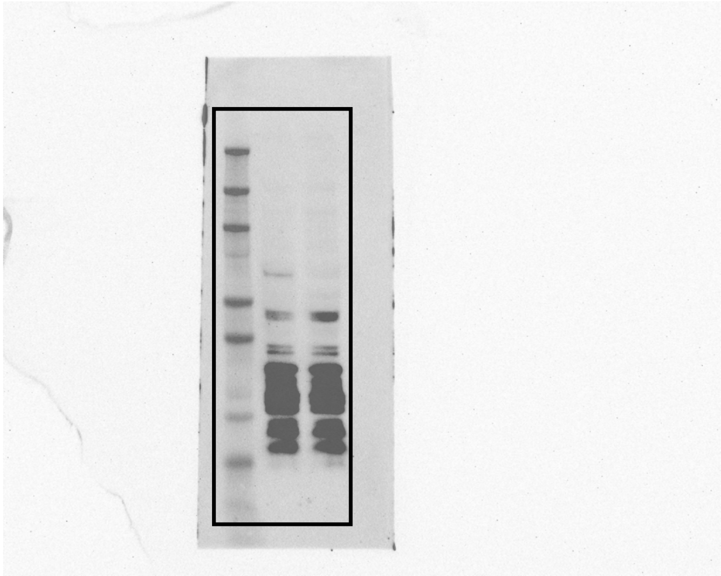

anti-PANX1-pY308
