# Supplementary material for: Human pannexin 1 channel is not phosphorylated by Src tyrosine kinase at Tyr199 and Tyr309
Source: eLife. 2024 May 23;13:RP95118. doi: 10.7554/eLife.95118 (PMC11115448; doi:10.7554/eLife.95118)
Supplement: Figure 4—source data 1. [file elife-95118-fig4-data1.zip › Figure 4-source data 1/figure 4 source data 1.pdf]

Figure 4-source data 1

Raw gel for Figurre 4A

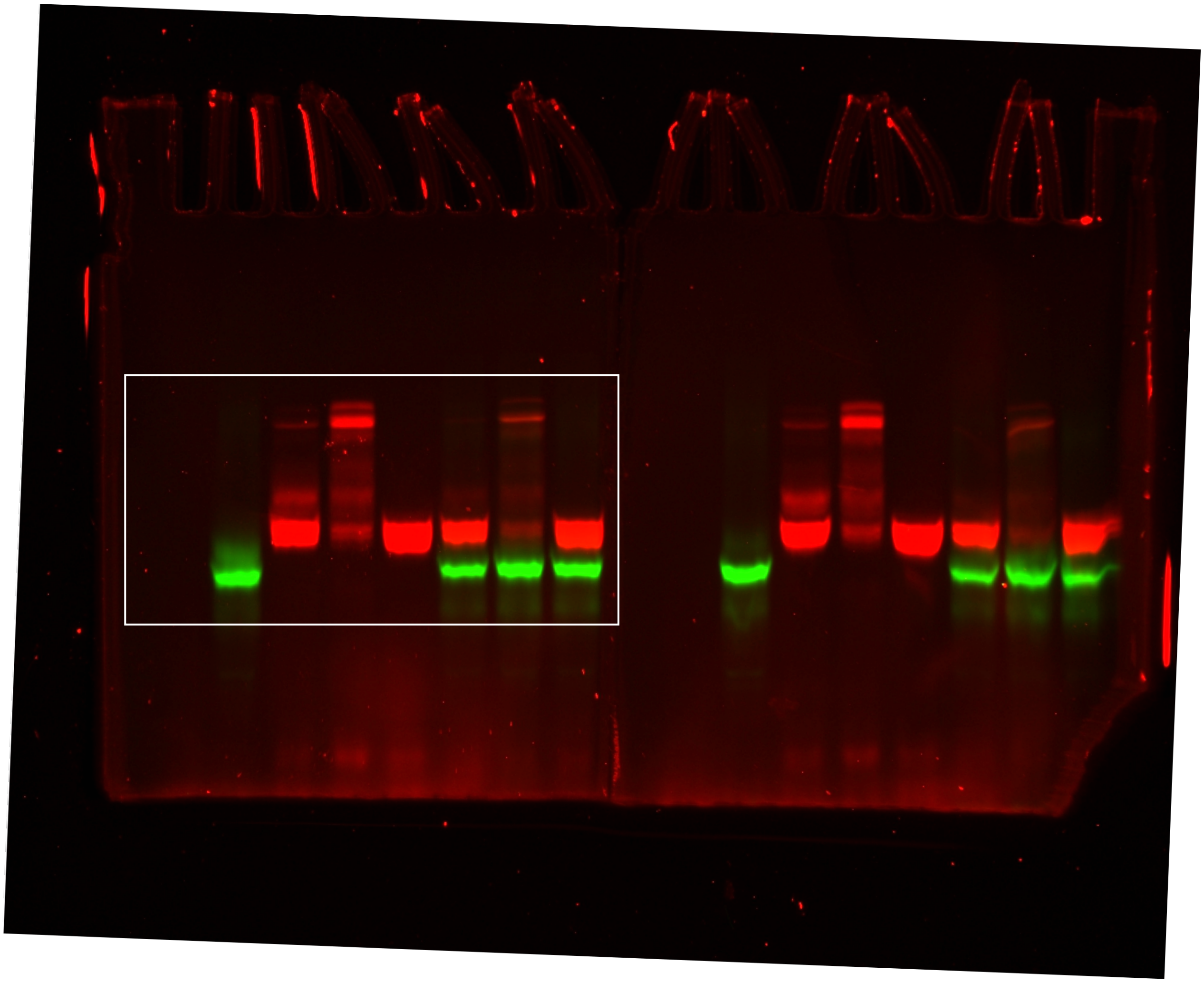

In-gel fluorescence

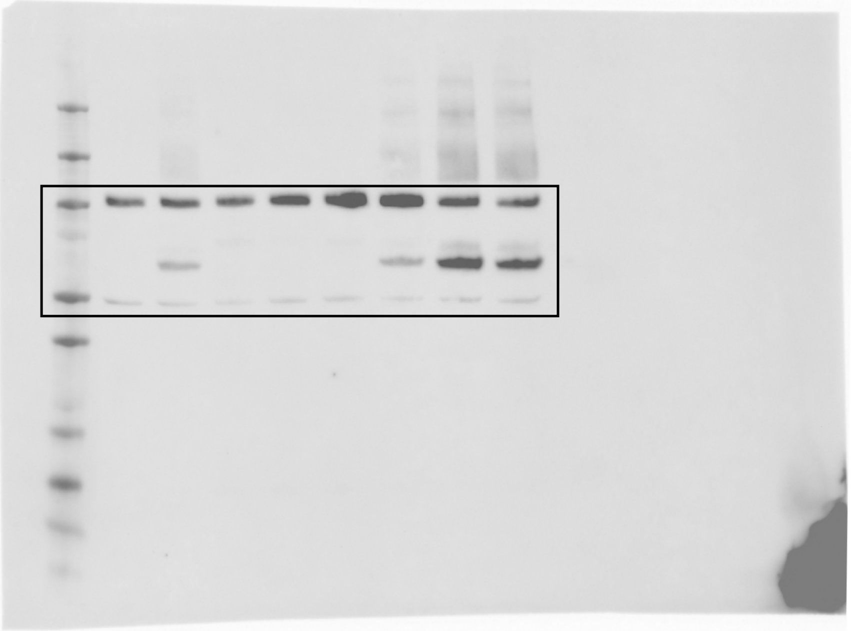

Upper panel of Figure 4B

anti-PANX1

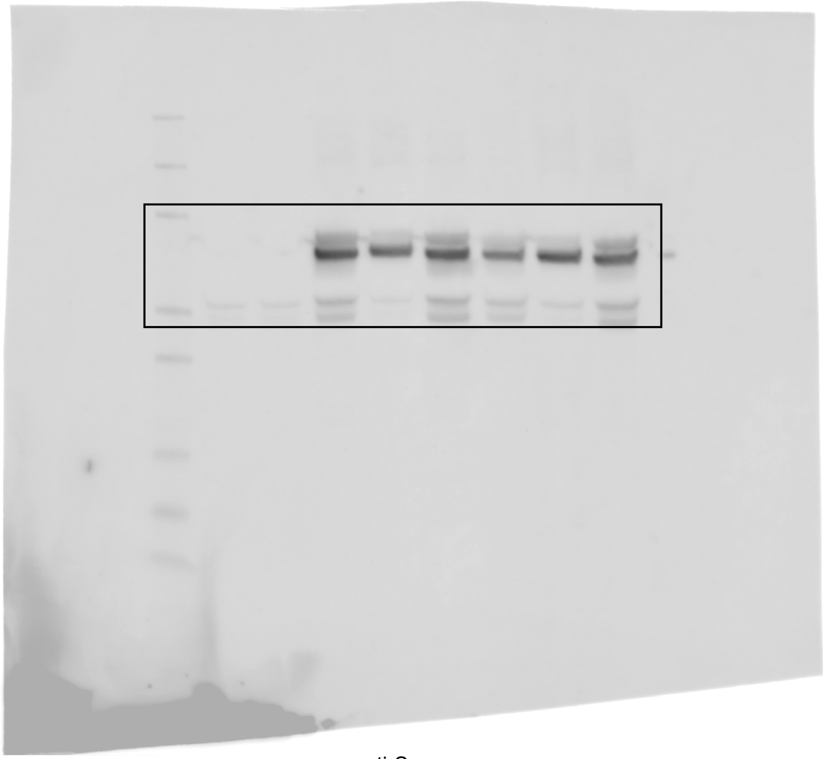

Middle panel of Figure 4B

anti-Src

Bottom panel of Figure 4B

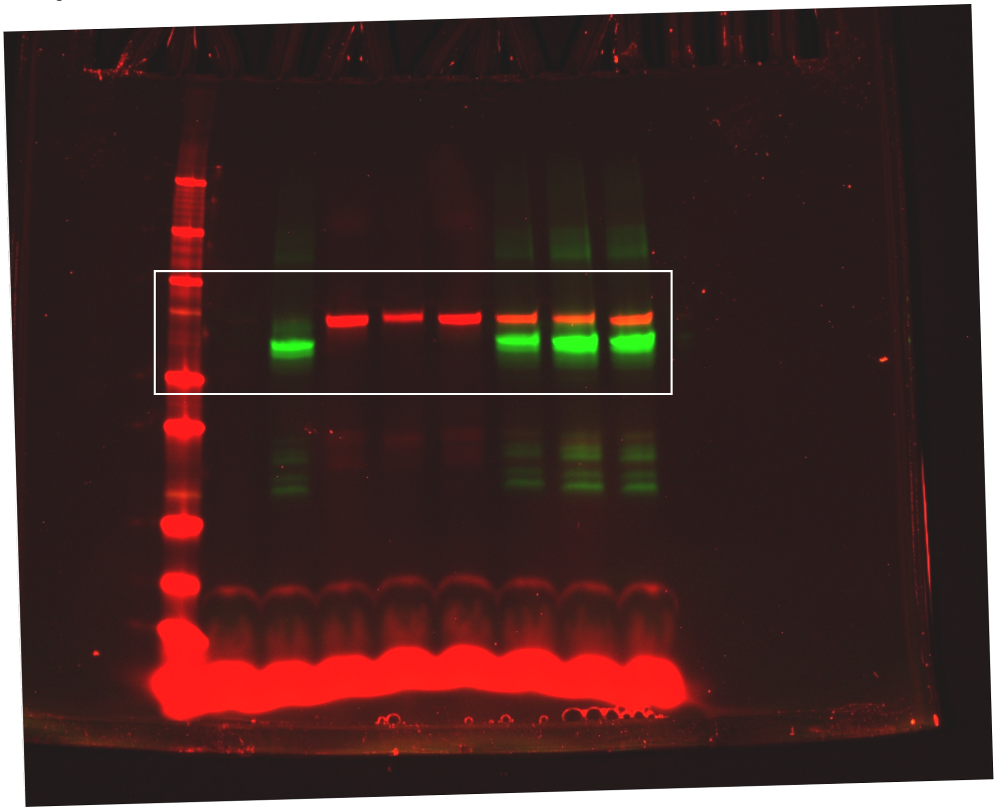

In-gel fluorescence
