# Supplementary material for: Human pannexin 1 channel is not phosphorylated by Src tyrosine kinase at Tyr199 and Tyr309
Source: eLife. 2024 May 23;13:RP95118. doi: 10.7554/eLife.95118 (PMC11115448; doi:10.7554/eLife.95118)
Supplement: Figure 4—figure supplement 2—source data 1. [file elife-95118-fig4-figsupp2-data1.zip › figure 4 figure supplement 2 source data 1/figure 4 figure supplement 2 source data 1]

Figure 4-figure supplement 2-source data 1

Upper panel of
Figure 4-figure supplement 2

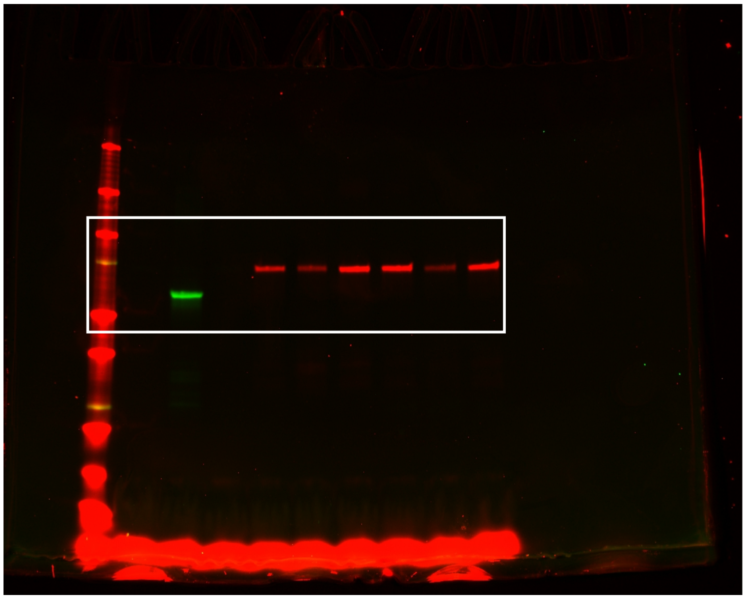

In-gel fluorescence

Middle panel of
Figure 4-figure supplement 2

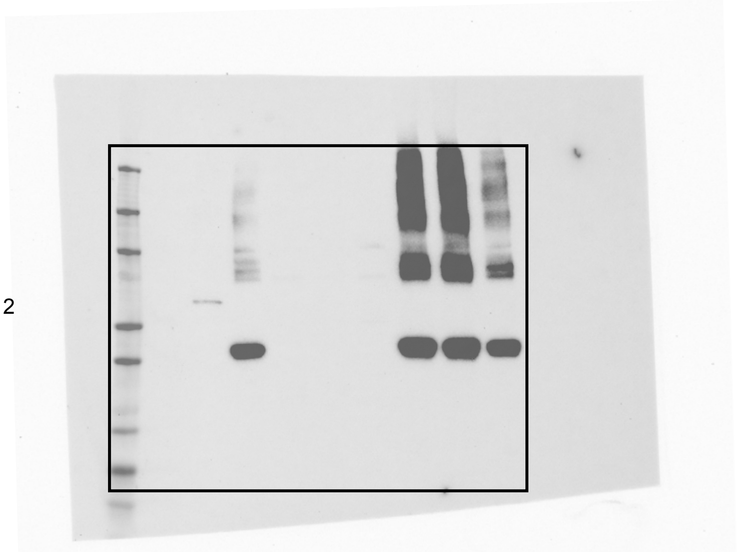

anti-PANX1

Lower panel of
Figure 4-figure supplement 2

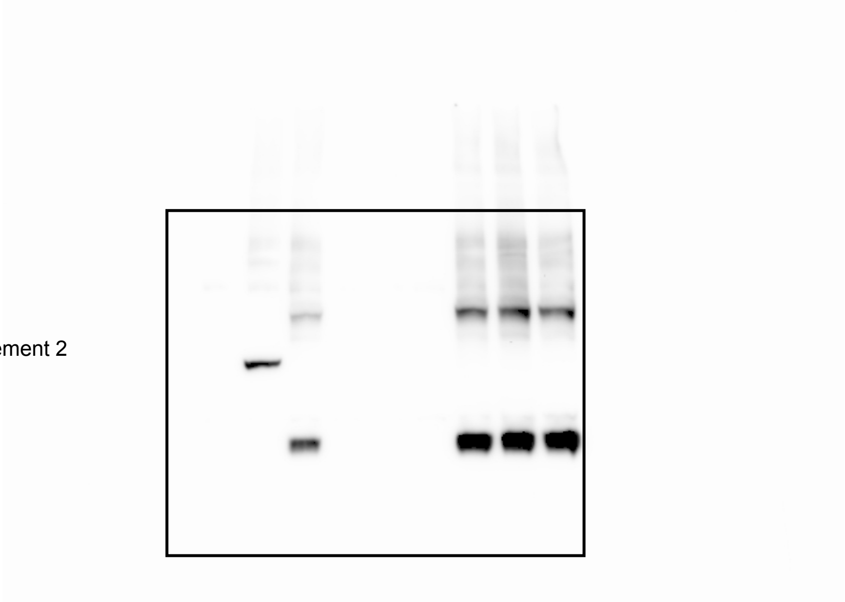

anti-PANX1
