# Supplementary material for: Human pannexin 1 channel is not phosphorylated by Src tyrosine kinase at Tyr199 and Tyr309
Source: eLife. 2024 May 23;13:RP95118. doi: 10.7554/eLife.95118 (PMC11115448; doi:10.7554/eLife.95118)
Supplement: Figure 5—source data 1. [file elife-95118-fig5-data1.zip › Figure 5 source data 1/figure5_source_data 1.pdf]

Figure 5-source data 1

Raw gel for Figure 5A

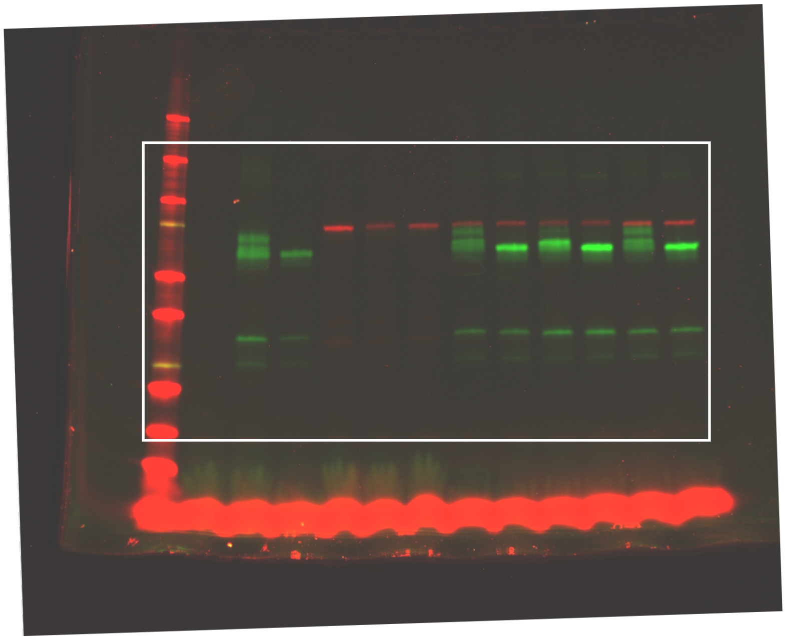

In-gel fluorescence

Raw gel for Figure 5B

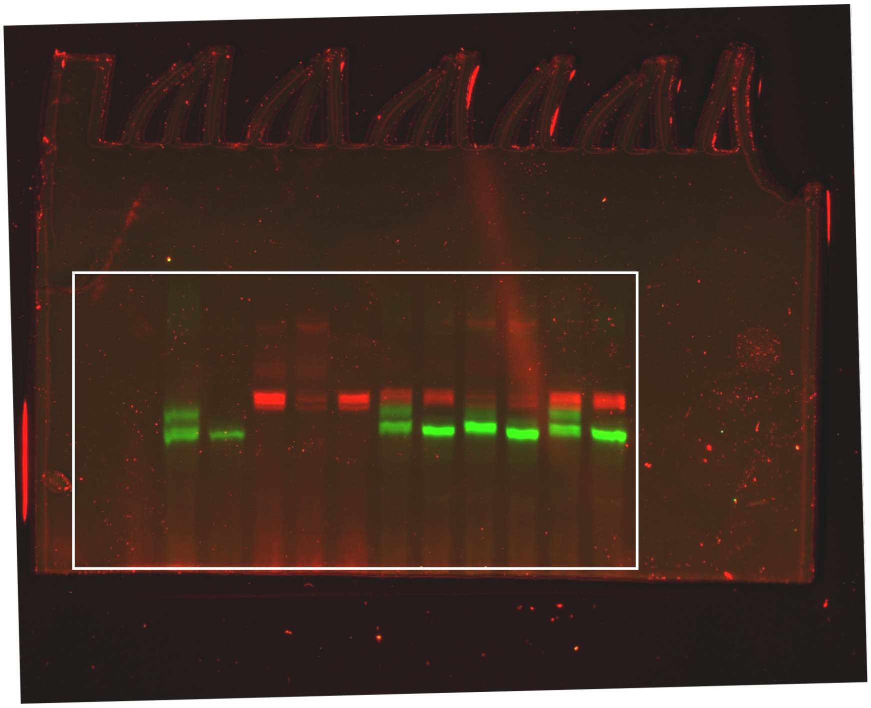

In-gel fluorescence
