# Supplementary material for: Human pannexin 1 channel is not phosphorylated by Src tyrosine kinase at Tyr199 and Tyr309
Source: eLife. 2024 May 23;13:RP95118. doi: 10.7554/eLife.95118 (PMC11115448; doi:10.7554/eLife.95118)
Supplement: Figure 6—source data 1. [file elife-95118-fig6-data1.zip › Figure 6 source data 1/figure 6 source data 1]

Figure 6-source data 1

Raw gel for Figure 6A

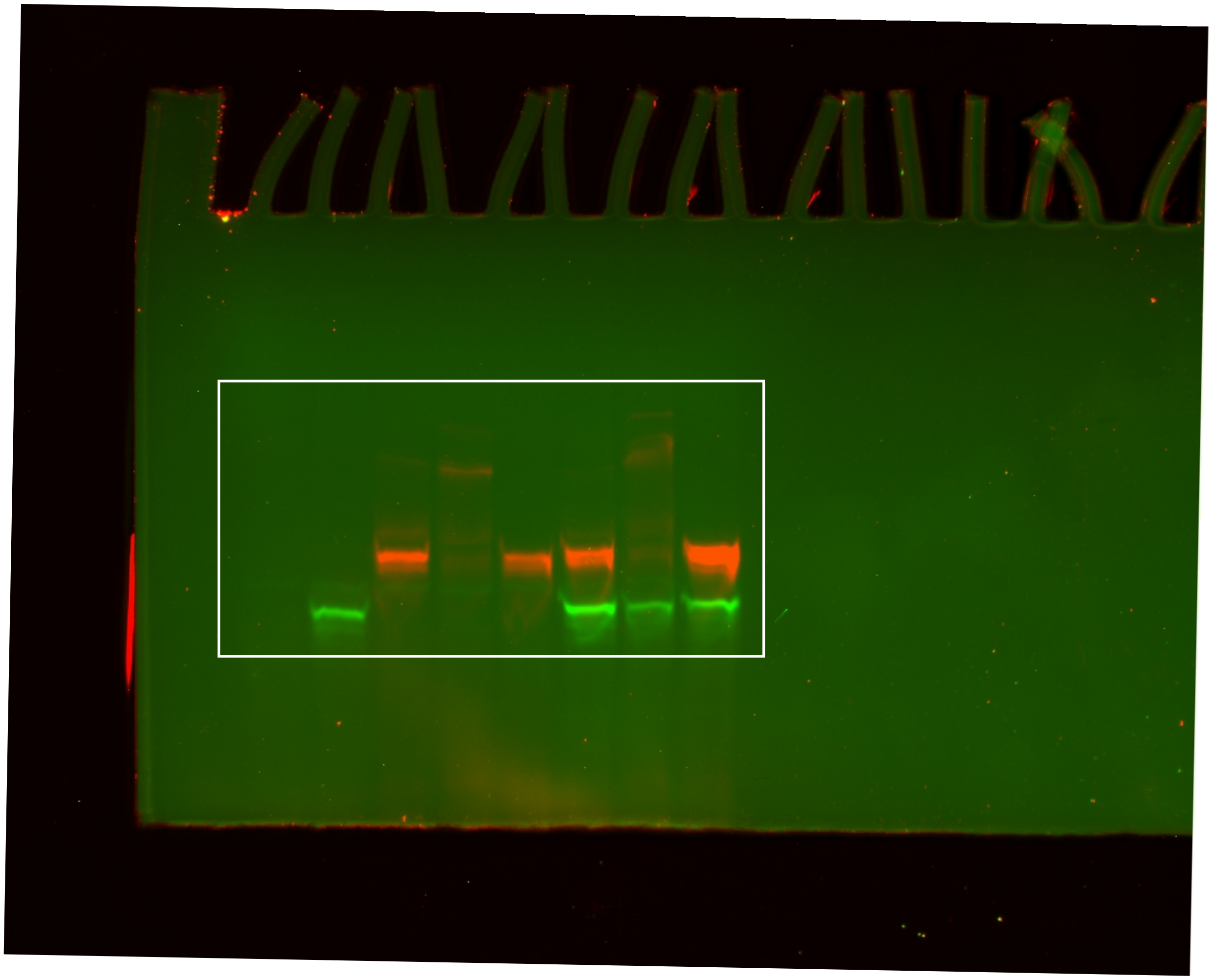

In-gel fluorescence

Upper panel of Figure 6B

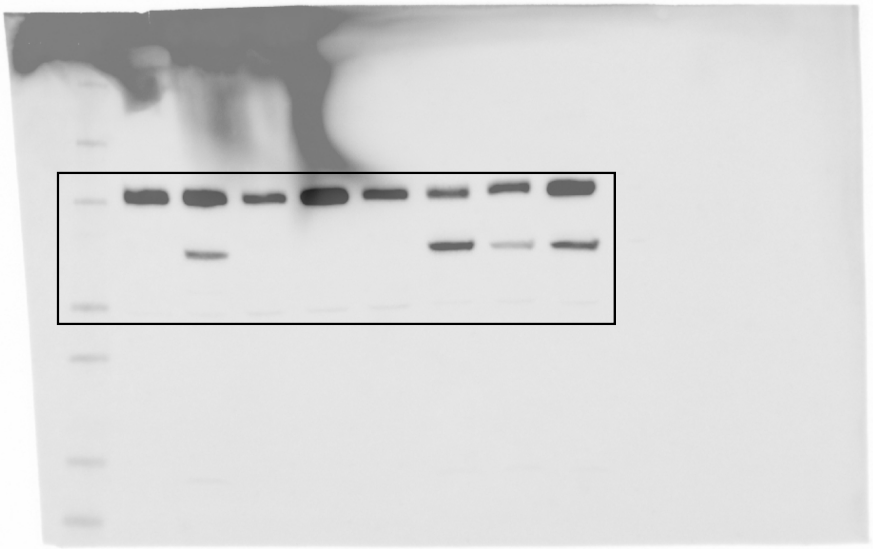

anti-PANX1

Middle panel of Figure 6B

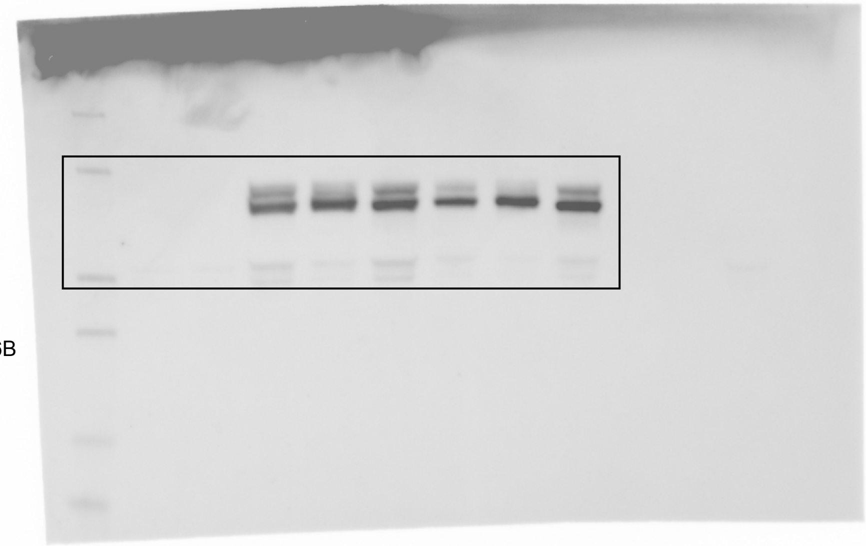

anti-Src

Bottom panel for Figure 6B

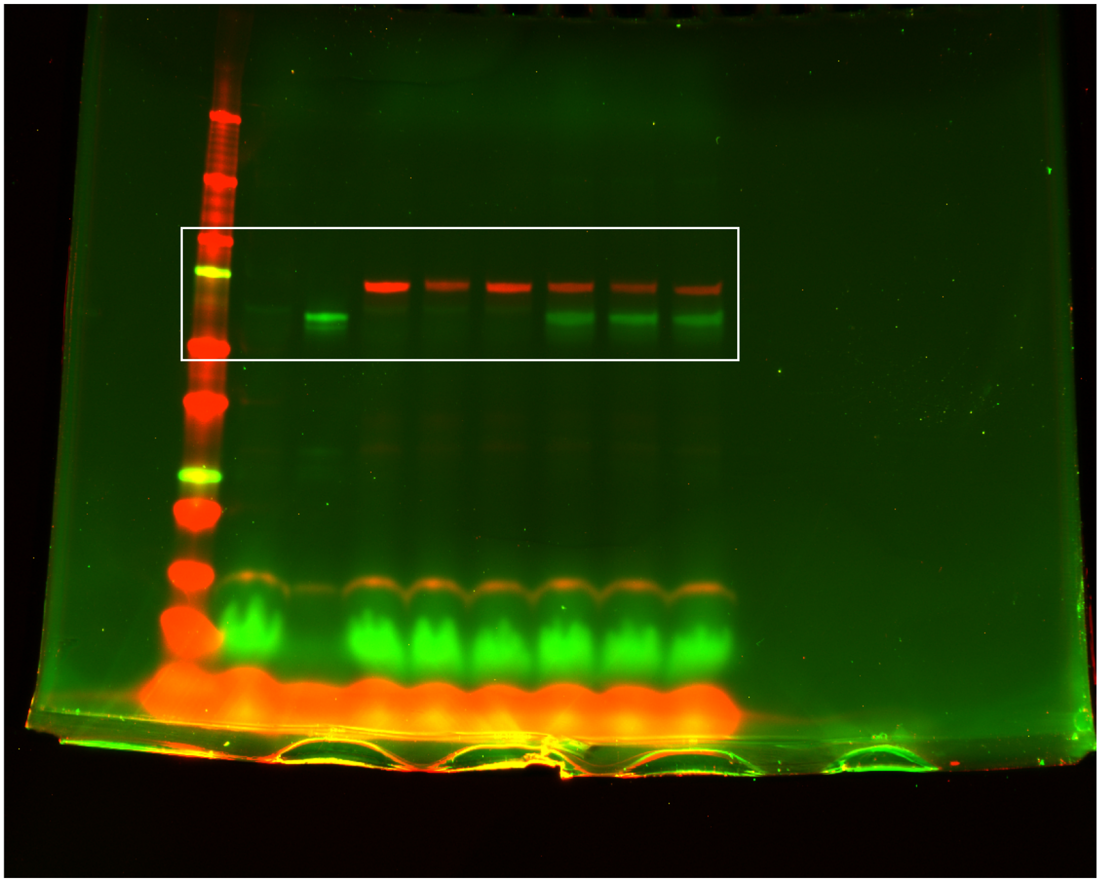

In-gel fluorescence
