# Supplementary material for: Human pannexin 1 channel is not phosphorylated by Src tyrosine kinase at Tyr199 and Tyr309
Source: eLife. 2024 May 23;13:RP95118. doi: 10.7554/eLife.95118 (PMC11115448; doi:10.7554/eLife.95118)

Figure 6-figure supplement 1-source data 1

Left panel of
Figure 6-figure supplement 1

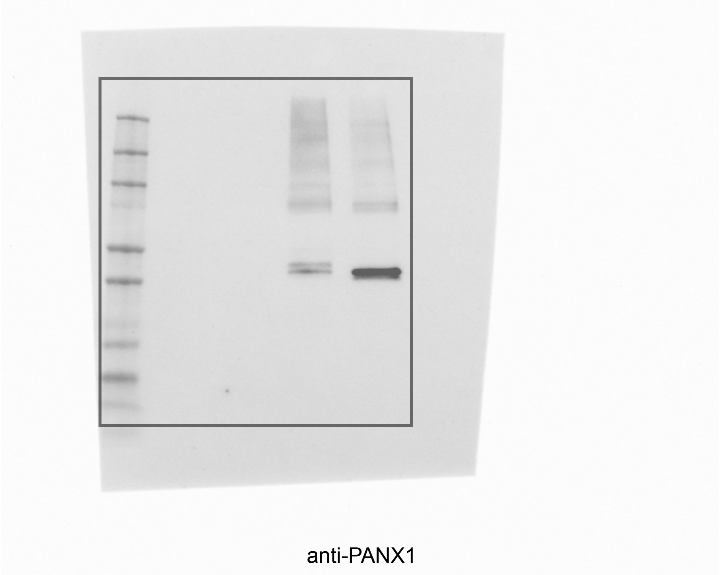

Right panel of
Figure 6-figure supplement 1

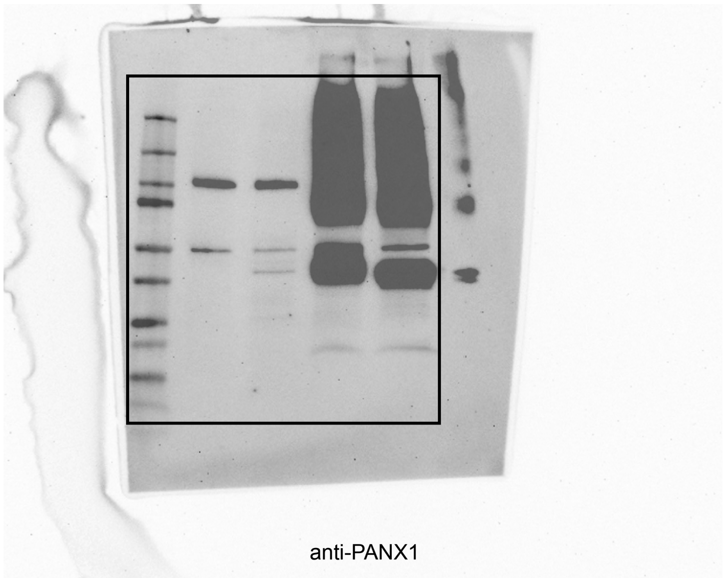

Supplement: Figure 6—figure supplement 1—source data 1. [file elife-95118-fig6-figsupp1-data1.zip › figure 6 figure supplement 1 source data 1/figure 6 figure supplement 1 source data 1]
